# Corticolimbic *DCC* gene co-expression networks as predictors of impulsivity in children

**DOI:** 10.1038/s41380-022-01533-7

**Published:** 2022-04-07

**Authors:** Jose M. Restrepo-Lozano, Irina Pokhvisneva, Zihan Wang, Sachin Patel, Michael J. Meaney, Patricia P. Silveira, Cecilia Flores

**Affiliations:** 1 Integrated Program in Neuroscience, McGill University, Montreal, QC Canada; 2 Douglas Mental Health University Institute, Montreal, QC Canada; 3 Ludmer Centre for Neuroinformatics & Mental Health, McGill University, Montreal, QC Canada; 4 Department of Psychiatry, Faculty of Medicine and Health Sciences, McGill University, Montreal, QC Canada; 5 Department of Neurology and Neurosurgery, Faculty of Medicine, McGill University, Montreal, QC Canada; 6 Singapore Institute for Clinical Sciences, Agency for Science, Technology and Research (A*STAR), Brenner Centre for Molecular Medicine, Singapore, Singapore

**Keywords:** Predictive markers, Genetics, Psychiatric disorders

## Abstract

Inhibitory control deficits are prevalent in multiple neuropsychiatric conditions. The communication- as well as the connectivity- between corticolimbic regions of the brain are fundamental for eliciting inhibitory control behaviors, but early markers of vulnerability to this behavioral trait are yet to be discovered. The gradual maturation of the prefrontal cortex (PFC), in particular of the mesocortical dopamine innervation, mirrors the protracted development of inhibitory control; both are present early in life, but reach full maturation by early adulthood. Evidence suggests the involvement of the Netrin-1/*DCC* signaling pathway and its associated gene networks in corticolimbic development. Here we investigated whether an expression-based polygenic score (ePRS) based on corticolimbic-specific *DCC* gene co-expression networks associates with impulsivity-related phenotypes in community samples of children. We found that lower ePRS scores associate with higher measurements of impulsive choice in 6-year-old children tested in the Information Sampling Task and with impulsive action in 6- and 10-year-old children tested in the Stop Signal Task. We also found the ePRS to be a better overall predictor of impulsivity when compared to a conventional PRS score comparable in size to the ePRS (4515 SNPs in our discovery cohort) and derived from the latest GWAS for ADHD. We propose that the corticolimbic *DCC*-ePRS can serve as a novel type of marker for impulsivity-related phenotypes in children. By adopting a systems biology approach based on gene co-expression networks and genotype-gene expression (rather than genotype-disease) associations, these results further validate our methodology to construct polygenic scores linked to the overall biological function of tissue-specific gene networks.

## Introduction

Several psychiatric disorders of developmental origin are characterized by deficits in cognitive control – a compromised ability to voluntarily choose a context-appropriate goal-directed response. Altered connections and communication between prefrontal and striatal regions appear to be at the core of this behavioral trait [1], but the underlying neurobiological processes, as well as early markers of vulnerability, are yet to be discovered [2–4]. The cognitive capacity to control and override impulsive behaviors improves gradually from childhood to early adulthood, mirroring the protracted developmental trajectory of the prefrontal cortex (PFC) [5–9], and its gradual quantitative and qualitative changes in dopamine innervation [10–12]. While dopamine axons establish local connections in the nucleus accumbens (NAcc) in adolescence, mesocortical dopamine axons are still growing from the NAcc to the PFC across this period [13–18]. The extent and organization of mesocortical dopamine axon growth in adolescence determines the organization of local PFC circuitry and cognitive function in adulthood [19–21].

The developmental trajectories of mesocortical and mesolimbic dopamine inputs are temporally different but have a reciprocal functional connection [21–24]. Transient postnatal developmental overexpression of dopamine D2 receptor in the striatum leads to adult mesocortical dopamine PFC dysfunction and cognitive deficits, indicating that striatal dopamine maturational events interact with those controlling mesocortical dopamine axon growth [25]. Changes in PFC dopamine neurotransmission are associated with opposite changes in NAcc dopamine function [23, 26], and alterations in D1- or D2-expressing NAcc pathways impact gene expression in the PFC [27]. Clearly, PFC and cognitive control development involve the recruitment of corticostriatal neuronal networks [28, 29].

A rapidly increasing number of studies show a strong association between genetic variability within the Netrin-1 guidance cue receptor gene, *DCC*, and several psychiatric disorders of developmental onset, most notably those emerging in adolescence. These disorders are characterized by PFC and NAcc dysfunction and deficits in impulse control [30–33]. Early postnatal expression of the *DCC* gene network in the PFC associates with total brain volume in children [34], emphasizing that the Netrin-1/*DCC* guidance cue system is tightly linked to overall early neurodevelopment. In adolescent rodents, DCC-mediated Netrin-1 signaling organizes the maturation of dopamine networks by promoting mesolimbic dopamine axon targeting in the NAcc and controlling the growth of dopamine axons to the PFC [12, 17]. Changes in DCC receptor levels in adolescent mice lead to mistargeting of mesolimbic dopamine axons in the NAcc and to their ectopic growth to PFC, altering PFC function and cognitive control in adulthood [17, 35]. Similar anatomical and behavioral changes occur in humans that are *DCC* mutation carriers [36, 37], indicating that the Netrin-1/*DCC* pathway is part of a gene network closely involved in corticolimbic development.

To date, most human genetic studies have focused on associations between genetic variants and phenotypes, and the estimated effects of a given number of variants can be aggregated into a score that represents individual genetic risk (called polygenic risk score; PRS). This association between genetic variation and behavior/disease ultimately results in relatively few genome-wide significant variants (e.g. [38]), most of which belong to noncoding portions of the genome and whose effect is diminished by the polygenic nature of complex phenotypes [39, 40]. Several of these non-coding variants are regulatory in nature, likely affecting the expression of nearby genes [40], ultimately placing gene expression as an intermediate molecular phenotype between genetics and disease [41]. Our approach exploits the facts that genes operate within complex networks, code with remarkable tissue-specificity for precise biological functions, and the likelihood of identifying relevant biological markers increases by relying on genotype-gene expression rather than genotype-disease associations (see [42, 43]). We use a systems biology approach to create a genetic score based on genes co-expressed with a gene of interest in a specific brain region. We gather all SNPs from the co-expressed genes and assign for each SNP the effect size estimated by the Genome-Tissue Expression (GTEx) project [44], which quantifies the influence of variants on tissue-specific gene expression. We aggregate genotypes weighted by the GTEx across all SNPs within the co-expression network into an expression-based polygenic score (ePRS), according to the individual’s genotype [42, 43].

The relationship between genes and behavior is highly indirect, regardless of how strong the relationship may be. Here, we forgo direct genotype-disease associations to construct an ePRS based on genes co-expressed with *DCC* in the PFC and the NAcc. Our goal is to create a marker that captures individual variation in the processes involved in the maturation of corticolimbic substrates supporting inhibitory control. By modifying the approach to genomic profiling, we generated a biological marker that can help identify early vulnerability for impulsivity-related phenotypes. We tested the association of the biologically-informed genetic score with measurements of impulsivity in three ethnically different community samples of 6- and 10-year-old children.

## Materials and methods

Detailed description is provided in the Supplementary Materials and Methods.

### Participants

We used genomic and phenotypic data from three prospective birth cohorts: (1) Maternal Adversity, Vulnerability and Neurodevelopment (MAVAN [45]), (2) Growing Up in Singapore Towards Healthy Outcomes (GUSTO[46]), and (3) Avon Longitudinal Study on Parents and Children (ALSPAC, detailed block diagram in Fig. S1 [47, 48]). Informed consent was obtained from each participant, and the the projects have been approved by: (1) McGill University, Université de Montréal, Royal Victoria Hospital, Jewish General Hospital, Centre hospitalier de l’Université de Montréal, Hôpital Maisonneuve-Rosemount, St Joseph’s Hospital, and McMaster University for MAVAN; (2) The National Healthcare Group Domain Specific Review Board and the Sing Health Centralized Institutional Review Board for GUSTO; and (3) the ALSPAC Ethics and Law Committee and the Local Research Ethics Committees. See Supplementary Table S6 for a summary of the genotyping information for each cohort.

### Identification of corticolimbic *DCC* gene co-expression networks and ePRS calculation

**Fig. 1:**
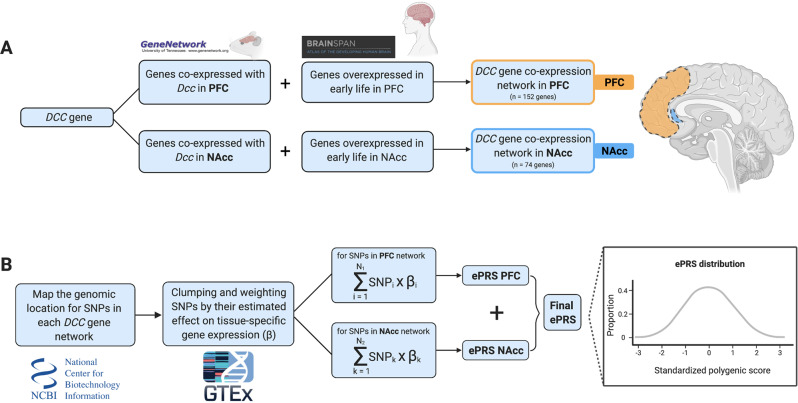
Flowchart depicting the steps involved in the creation of the corticolimbic *DCC*-ePRS score. **A** The GeneNetwork database was used to generate a *Dcc* gene co-expression matrix in the PFC and NAcc in mice. Genes with a correlation of co-expression $\ge$|0.5| were retained. Brainspan was used to identify human homologous transcripts and to filter each gene list by selecting the transcripts enriched during the first 18 months of life, as compared to adulthood, defined by a differential expression $\ge$1.5, within the same brain area. Each resulting gene list comprised the *DCC* co-expression network for their respective brain area. **B** Based on their annotation in the NCBI library, using GRCh37.p13 assembly, common SNPs within each co-expression network, GTEx data base, and genotyping cohort were subjected to linkage disequilibrium clumping to remove highly correlated SNPs (*r*^2^ $\ge$ 0.2). Using data from the GTEx project, alleles at a given cis-SNP were weighted by the estimated brain-region-specific effect of the genotype on gene expression. The sum of these estimated effects resulted in ePRS scores for the *DCC* co-expression networks in the PFC and NAcc, which we aggregated into a single global ePRS score.

Figure 1 shows the steps involved in the identification of the gene networks and the ePRS score. The ePRS was calculated considering genes co-expressed with *DCC* in the PFC and NAcc. We aimed to capture *DCC* co-expression networks within each brain region, with the final ePRS being a joint representation of the functional co-expression networks in these two corticolimbic regions. As described previously [42, 43, 49], the score was created using the data from: (1) GeneNetwork (http://genenetwork.org), (2) BrainSpan (http://www.brainspan.org), (3) the NCBI Variation Viewer, U.S. National Library of Medicine, (NCBI) [50], (4) the GTEx project [44], and (5) genotype data in the three cohorts. We used GeneNetwork to generate a list of genes co-expressed with *DCC* in the PFC and in the NAcc in mice (absolute value of co-expression correlation greater or equal to 0.5). We used gene expression datasets from mice (see Supplementary Data file) because our study is guided by our previous findings in rodents linking variations in *Dcc* expression to changes in impulse control and in mesocorticolimbic dopamine axon targeting [13, 17, 51, 52]. To retain genes that are more active when the brain is still undergoing core maturational processes in humans, we used BrainSpan to select autosomal transcripts expressed at least 1.5-fold more during the early postnatal development (0–18 months after birth) than in adulthood (20–40 years of age), with the final networks consisting of 154 genes in the PFC (see Table S4) and 72 genes in the NAcc (see Table S5). For annotations, we used GRCh37.p13 assembly of the NCBI to source chromosome and start/end position for the co-expressed genes, which, in turn, were used to gather all the gene-SNP pairs from the GTEx dataset in human PFC and NAcc (PFC: 41,053 SNPs, NAcc: 66,428 SNPs). These lists were merged with the genotyping data in each of the three cohorts, keeping only the common SNPs and subjecting the final genotyping data sets to linkage disequilibrium clumping (r^2^ < 0.2) to eliminate highly correlated SNPs.

To calculate the ePRS, number of effect alleles at a given cis-SNP were weighted by the estimated brain-region-specific effect of the genotype on gene expression from the GTEx data. The ePRS was obtained by adding the weighted SNPs, accounting for the sign of the correlation between each gene’s expression and *DCC* gene expression. The sum of the estimated effects resulted in ePRS scores for the *DCC* co-expression networks in the PFC and NAcc, which were then aggregated (by summation of the two scores) into a single global genetic score termed “corticolimbic *DCC*-ePRS”.

Finally, an enrichment analysis was conducted to characterize the functional and biological properties of the gene networks that comprise the corticolimbic *DCC*-ePRS score. A description of the tools and datasets used throughout the study can be found in the Supplementary Material, Table S7.

### Behavioral outcomes

We tested whether the ePRS associates with two aspects of impulsivity: (i) impulsive choice, reflecting proneness to make risky choices, as measured by the Information Sampling Task; (ii) impulsive action, reflecting the ability to inhibit motor responses, as measured by the Stop-Signal Task. In both cases, the ability to self-regulate behavior is required for interrupting or inhibiting competing inputs or actions in order to accomplish a specific goal-directed response [1]. A description of the behavioral data obtained from each cohort is described in the Supplementary Material.

### Statistical analysis

Data were analyzed using R v3.6 [53] and Python v3.7 (https://www.python.org/), and polygenic scores were generated using the PRSoS pipeline (https://github.com/MeaneyLab/PRSoS). We considered two-tailed hypothesis tests, and significance levels for all tests were set at α < 0.05. For each cohort we categorized the ePRS into high- and low-ePRS groups using a median split of the genetic score. Analysis of baseline characteristics was performed using Student’s *t*-test for continuous data (in case of unequal variances, Welch’s *t* test was used) and X^2^ for categorical variables. Linear regression analysis was used to examine the association of the ePRS with the behavioral outcomes, adjusting for sex and population stratification. Adjustment for multiple comparisons was applied using Bonferroni method, independently for each behavioral construct/cohort. All data were inspected to ensure that the assumptions for the tests and the linear regression analyses were met. Power analysis was conducted for linear multiple regression, considering the effect of the ePRS on the different outcomes: for α = 0.05, sample size of 202, 398, and 4392, and a small effect size f^2^ = 0.02, the achieved power will be 0.64, 0.88, and > 0.95 in MAVAN, GUSTO, and ALSPAC, respectively.

## Results

We found no differences in baseline characteristics between ePRS groups in MAVAN, GUSTO, and ALSPAC cohorts (Table 1).

**Table 1:** Description of baseline characteristics of the 3 cohort samples.

**MAVAN**				
*Sample description*	*Total (n* = *202)*	*Low ePRS (n* = *96)*	*High ePRS (n* = *106)*	*p-value*
Sex – male (*n*)	49.5% (100)	56.3% (54)	43.4% (46)	0.09
Maternal age at birth (years)	30.72 (4.90)	31.42 (5.07)	30.08 (4.67)	0.053
Gestational age (weeks)	39.03 (1.23)	38.89 (1.30)	39.16 (1.15)	0.11
Birth weight (g)	3313 (452)	3300 (450)	3224 (456)	0.71
Maternal education – University degree or above	55.7% (108)	54.3% (50)	56.9% (58)	0.47
Low family income	20.6% (50)	25.8% (23)	27.3% (27)	0.96
**GUSTO**				
*Sample description*	*Total (n* = *398)*	*Low ePRS (n* = *202)*	*High ePRS (n* = *196)*	*p-value*
Sex – male (*n*)	53.3% (212)	49.5% (100)	57.1% (112)	0.15
Maternal age at birth (years)	31.55 (5.04)	31.81 (5.34)	31.28 (4.73)	0.37
Gestational age (weeks)	38.49 (1.28)	38.47 (1.33)	38.50 (1.23)	0.87
Birth weight (g)	3137 (416)	3127 (441)	3147 (391)	0.39
Maternal education – University degree or above	35.64% (103)	38.36% (56)	32.88% (47)	0.39
Household income < $2000 SGD per month	12.11% (35)	15.75% (23)	8.39% (12)	0.08
**ALSPAC**				
*Sample description*	*Total (n* = *4392)*	*Low ePRS (n* = *2210)*	*High ePRS (n* = *2182)*	*p-value*
Sex – male (*n*)	49.2% (2159)	48.6% (1075)	49.7% (1084)	0.51
Maternal age at birth (years)	29.31 (4.47)	29.31 (4.47)	29.31 (4.47)	0.97
Gestational age (weeks)	39.76 (1.27)	39.79 (1.26)	39.73 (1.28)	0.11
Birth weight (g)	3499 (465)	3503 (458)	3496 (471)	0.61
Maternal education – University degree or above	18.9% (786)	19.7% (411)	18.2% (375)	0.25
Low SES^a^	35.0% (1537)	34.5% (762)	35.5% (775)	0.49

Continuous variables are expressed as mean (SD); categorical variables are expressed as percentage (number of subjects).

^a^We used “crowding index” as a proxy measure for SES. This index was calculated by dividing the number of individuals living in the family’s residence, by the number of rooms in the residence, and we considered low SES when crowding index > 0.75, and high SES when crowding index = 0.75.

### Behavioral outcomes

#### Lower Corticolimbic *DCC*-ePRS Scores Associate with Higher Measurements of Impulsivity in Children

**Fig. 2:**
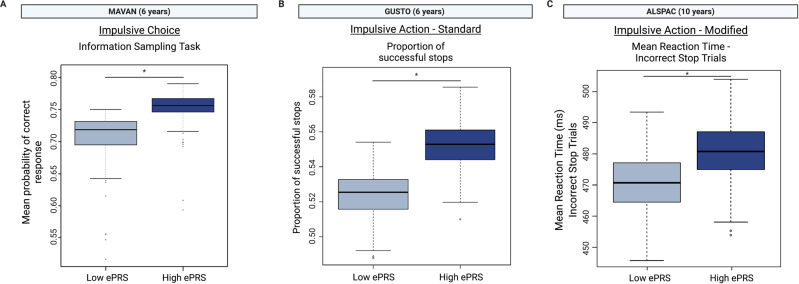
Association between ePRS scores and impulsivity-related phenotypes. Association between the corticolimbic *DCC*-ePRS score and measurements of impulsivity, in (**A**) MAVAN kids (*n* = 197) at 6 years of age (Information Sampling Task: $\beta$ = −0.04, *p* = 0.045), (**B**) GUSTO kids (*n* = 398) at 6 years of age (Proportion of successful stops: $\beta$ = −0.03, *p* = 0.027), and (**C**) ALSPAC kids (*n* = 4392) at 10 years of age (Mean RT – Incorrect stop trials: $\beta$ = −10.36, *p* = 0.019). A lower *DCC*-ePRS score was associated with higher impulsive action and choice in the 3 ethnically-diverse cohorts. The *Y* axis represents the predicted values of the measurements of impulsivity, the middle of the box is the median, the edges are the lowest and highest quartiles, and the error bars (whiskers) represent 1.5 x IQR (interquartile range). *p < 0.05.

Information Sampling Task: In MAVAN, the ePRS was associated with meanP-correct values (Fig. 2A: $\beta$ = −0.04, *p* = 0.045); the low-ePRS group had lower meanP-correct values (less information sampled, lowering the probability of making a correct choice at the point of decision) indicating higher levels of impulsive choice in comparison to the high-ePRS group.

Stop-Signal Task: In GUSTO, the ePRS was associated with the proportion of successfully inhibited responses (Fig. 2B: $\beta$ = −0.03, *p* = 0.027; Fig. S3 for complete results); low-ePRS group has a lower proportion of successful stops compared to the high-ePRS group, indicating higher levels of impulsive action. In ALSPAC, the ePRS was associated with measurements of impulsive action (Fig. 2C: $\beta$ = −10.368, *p* = 0.019; Fig. S4 for complete results), with the low-ePRS group showing a shorter mean reaction time in unsuccessful stop trials- indicating higher levels of impulsive action- compared to the high-ePRS group. Also in ALSPAC, there are no differences between ePRS groups when comparing the proportion of successful stops, but methodological differences (full details in supplementary methods; see Fig. S2) in the way the task was conducted in CANTAB (“stop” signal delay was adjusted to subject’s performance in MAVAN and GUSTO) versus ALSPAC (fixed delay of 250 ms was applied irrespective of subject’s performance) prevent the direct comparison of successfully inhibited responses between these cohorts. In MAVAN, we found no association between the ePRS and performance in this task (Proportion of successful stops: $\beta$ = −0.03, *p* = 0.10; Estimated SSRT: $\beta$ = −10.07, *p* = 0.57; see Table S2 for complete results). After adjustments for multiple comparisons, the association between the ePRS and the proportion of successful stops in GUSTO cohort is no longer significant (*p* = 0.054).

These results show that an ePRS score reflecting variability in the expression of corticolimbic *DCC* gene co-expression networks is associated with the levels of inhibitory control in children from ethnically diverse backgrounds.

### *Corticolimbic DCC* gene co-expression networks: enrichment analysis

#### Protein–protein interaction (PPI)

**Fig. 3:**
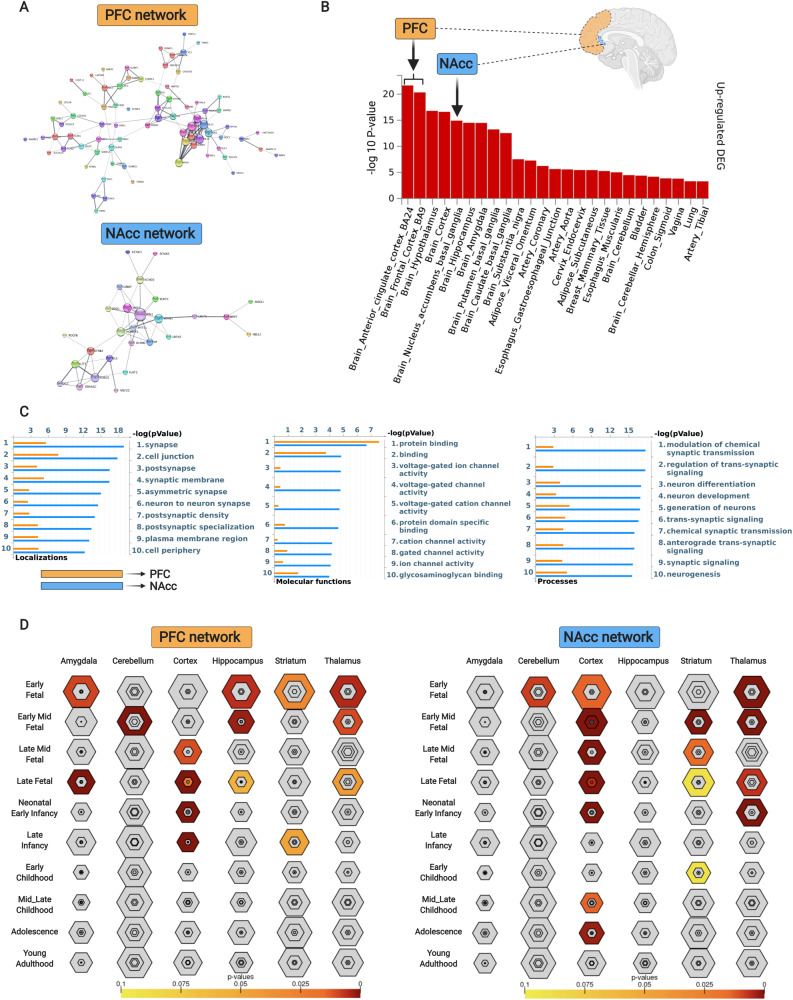
Validation of the PFC and NAcc *DCC* co-expression networks. **A** Protein–Protein interaction (PPI) networks constructed from the gene co-expression networks in PFC and NAcc, using the Cytoscape software. The edges between the nodes indicate both functional and physical associations, and the size of the sphere is proportional to the degree of connectivity with other nodes. The protein networks represent known functional interactions between the protein products of the genes that make up the corticolimbic *DCC* gene networks. Significant PPI enrichment in the PFC (*p* = 0.004) and the NAcc (*p* = 5.1e-11). **B** Tissue-specific gene expression analysis performed in FUMA confirms that the genes that comprise both networks are highly upregulated in the PFC and NAcc, according to GTEx dataset v8. **C** A combined enrichment analysis for the co-expression networks performed in Metacore ^TM^ shows enrichment for diverse neurodevelopmental processes, suggesting a common brain maturational role for the networks (see full results with FDR adjusted values in Table S3). **D** Cell-type Specific Expression Analysis (CSEA) analysis reveals that the NAcc and PFC *DCC* co-expression networks are highly enriched throughout the brain during embryonic life and early infancy. However, the NAcc network is enriched again in the cortex during late childhood and adolescence (*p* = 0.0004 for Fisher’s exact test, *p* = 0.002 after Benjamini-Hochberg correction). The hexagon levels mark the different degrees of stringency applied in the identification of selectively enriched transcripts for that brain region/developmental period. In each hexagon there are 4 levels, with the outer level representing the least stringent pSI value (0.05) and the inner-most level consisting of the most stringent pSI value (0.0001). The 2 hexagons in between represent a pSI = 0.01 and a pSI = 0.001. The size of the hexagon is proportional to the number of genes selectively enriched, and the color represents the FDR-adjusted *p* values of the expected overlap between the genes in the network and the list of selectively enriched genes.

We used STRING [54] and Cytoscape [55] to visualize catalogued PPIs in protein products of genes within each co-expression network (networks were analyzed separately; only proteins with interactions are depicted in Fig. 3A). PFC: This network contains 152 nodes (one for each protein with at least 1 connection with another protein in the network) and 151 edges, corresponding to the mapped interactions among the nodes. The PPI enrichment (*p* = 0.004) indicates that this network contains more interactions than expected, compared to a network of equal size composed of a random set of proteins, and that the proteins are involved in common biological functions. NAcc: Contains 74 nodes and 50 edges, and the PPI enrichment (*p* = 5.1e-11) also suggests a strong biological connection among the proteins (see the corresponding gene networks, created using GeneMANIA [56], in Fig. S5).

#### Tissue-specific gene expression

We used FUMA [57] to visualize the expression levels of the genes from the co-expression networks across the 54 tissue-types included in GTEx. The PFC (comprising BA24 and BA9) and the NAcc are the 1st and 4th most enriched tissues for the gene networks’ expression (Fig. 3B).

#### Functional ontologies

Using Metacore^TM^, we explored the biological context in which the gene networks operate, by mapping the genes from each network onto functional categories (Fig. 3C, Table S1 for more detailed results). The networks are enriched in synaptic components, predominantly in cell junction and plasma membrane regions (strongest enrichment for cell junction in PFC: *p* < 3.3e-8, and for synapse in NAcc: *p* < 1.3e-19). Enrichment for biological processes showed the involvement of the network in neurodevelopmental processes including neuronal differentiation and development (PFC: *p* < 2.1e-4; NAcc: *p* < 1e-19), neuron projection guidance (PFC: *p* < 1e-3; NAcc: *p* < 1e-16) and regulation of trans-synaptic signaling (PFC: *p* < 2.9e-5; NAcc: *p* < 3.09e-17). Enrichment for molecular functions showed a role of the networks in protein binding and cell adhesion (PFC: *p* < 2.72e-8; NAcc: *p* < 5.6e-6). These functions are fundamental to the establishment of brain connectivity, mainly via axon guidance (e.g. [58]) and synaptogenesis (e.g. [59]).

#### Developmental gene expression

We assessed the enrichment of gene expression for each network across brain regions and developmental periods in humans, using the CSEA tool [60, 61]. Both networks are enriched across the brain during perinatal periods. Notably, the expression of the NAcc network in the PFC is enriched again during adolescence (Fig. 3D: *p* = 3.679e-04), in line with previous descriptions of the developmental trajectory mediating adolescent corticolimbic maturation [17, 25].

**Fig. 4:**
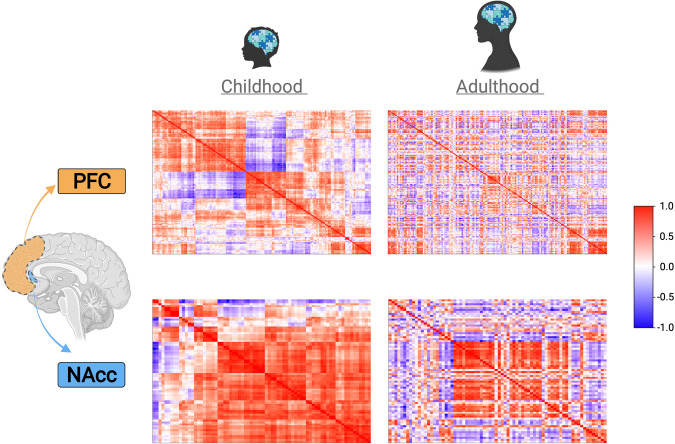
Co-expression of the genes in the corticolimbic *DCC* ePRS across development in human PFC and NAcc. *Top panel*, PFC: The heatmap of the co-expression in childhood (left) shows several clusters, while for the co-expression patterns in adulthood (right) most of the clusters are not maintained, suggesting that genes that are co-expressed during childhood in the PFC are rarely co-expressed in adulthood. *Bottom panels,* NAcc: The heatmap in childhood (left) shows many clusters with a very high correlation of expression. Interestingly, a larger proportion of these clusters are maintained in adulthood (right) compared to the transition between childhood and adulthood in the PFC, indicating a more stable gene network. We retained the same order for the genes as in childhood, to be able to compare if the clusters that we observe in childhood are maintained in adulthood.

To explore the ability of the PFC and NAcc networks, and ultimately the ePRS itself, to capture transcriptionally co-regulated biological processes, we analyzed the networks’ co-expression patterns in their corresponding brain regions during childhood and adulthood using the Brainspan dataset ([62]; Fig. 4). PFC: In the heatmap representing correlation of gene expression during childhood, there are 3 main clusters of high co-expression, but only 1 cluster is maintained in adulthood. Finding that correlations of expression of genes in the PFC in childhood are not maintained in adulthood is in line with the marked developmental changes in the PFC transcriptome landscape previously described in humans and mice [63]. NAcc: The heatmap for childhood gene co-expression shows a large main cluster, containing several highly correlated smaller clusters. Many of the smaller NAcc clusters perdure into adulthood, indicating that the NAcc network is more stable than the PFC network.

### Comparison between polygenic scores

We compared our ePRS to a traditional PRS for ADHD on the capacity to predict the same behavioral outcomes. For that, we selected the top 4515 most significant SNPs identified in the latest ADHD GWAS [64], which corresponds to the GWAS *p*-value threshold 4.912e-5, and created a score comparable in size to the ePRS in terms of number of SNPs. There was no association between the PRS and the main outcomes for MAVAN (meanP-correct: $\beta$ = −0.01, *p* = 0.48), GUSTO (proportion of successful inhibitions: $\beta$ = −0.009, *p* = 0.52; Fig. S3) or ALSPAC (mean reaction time – incorrect stop trials: $\beta$ = −3.248, *p* = 0.46; Fig. S4) cohorts. We also performed an enrichment analysis to characterize the functional/biological properties of the PRS genes and found that they are upregulated across the brain, but not as selectively- and to a lesser extent- than the genes from the ePRS. Finally, results from the CSEA show no selective spatiotemporal enrichment in the human brain (Fig. S6).

## Discussion

Impulse control deficits are a common trait of numerous neurodevelopmental psychiatric disorders. Discovering their neurobiological underpinnings and early biomarkers will help identifying at risk individuals and improving/implementing early prevention and intervention strategies. Here, we generated an expression-based polygenic score (ePRS) consisting of SNPs within genes co-expressed with the axon guidance cue receptor gene, *DCC*, in the PFC and NAcc, to create a functional and corticolimbic-specific marker of vulnerability to heightened impulsivity. Our results show that the ePRS is significantly associated with different measures of impulsive behaviors in children from three ethnically diverse independent birth cohorts. Across all cohorts, the low-ePRS groups show higher impulsivity-related phenotypes. Detailed characterization of the gene networks comprising the corticolimbic *DCC*-ePRS show significant functional interactions, contribution to core neurodevelopmental processes, and enriched expression in cortical neurons, particularly from embryonic life to adolescence.

Most PRSs are characterized by a limited generalizability due to a marked disparity in prediction accuracy across different populations [65, 66]. This limitation, partially explained by the biased ancestry representation in most well-powered discovery GWASs, does not affect the ability of the ePRS to predict impulsive phenotypes across 3 independent birth cohorts from Canada, Singapore, and UK. Other studies that have implemented a similar approach to polygenic risk analysis by using the ePRS methodology reported a high predictive value of their genetic scores applied across diverse populations [42, 67]. To understand how the ePRS compares to a traditionally derived PRS, in this study we constructed a score based on the latest GWAS for ADHD and found that, even though the PRS score predicts impulsive behavior in one cohort (Fig. S3), our ePRS predicts a larger number of outcomes, across all three cohorts. This is consistent with other studies that have observed a higher prediction accuracy of their ePRS compared to conventional PRSs [34, 42]. Since the ePRS methodology relies on identifying tissue-specific gene networks and their function, instead of identifying scattered genetic variants across the genome, we are able to create a more biologically meaningful score compared to conventional PRSs. Finally, the associated phenotype to weigh the SNPs in our ePRS is gene expression, which, given the current state of technology, is a highly quantitative trait measurable with high precision, across different tissues and conditions, by high-throughput sequencing, and thus yielding a score that globally represents transcriptionally co-regulated biological processes. These results suggest that our genetic profiling approach increases the likelihood of identifying trait-relevant biological markers.

We identified co-expression networks for the guidance cue receptor, *DCC*, specifically in the NAcc and the PFC, to create a biological marker related to neurodevelopmental processes occurring in these regions, that could predict levels of impulsivity in children. In addition to establishing the ePRS’ predictive power of impulsivity across 3 different cohorts, we found the co-expression networks to be highly enriched for protein-protein interactions, suggesting their involvement in common biological functions. Since DCC receptors are master organizers of neuronal circuits, and since variations in its expression in early life result not only in functional and anatomical alterations of neural pathways involved in inhibitory control, but also in alterations of inhibitory control itself [17, 36, 51], it is not surprising that *DCC* co-expression networks in these corticolimbic hubs associate with behavioral traits implicated in psychopathology. Indeed, proper establishment of neuronal circuits is essential to mental health [68]. The genes that make up the networks are highly upregulated in the PFC and the NAcc and are involved in a wide range of neurodevelopmental processes. This enrichment suggests a prominent role of the gene networks in the maturation of both PFC and NAcc circuits, validating the use of these networks as the basis for the ePRS calculation and their potential use as a functional biomarker to predict reflection and motor impulsivity in children.

Results from several studies in humans show that mutations in the *DCC* gene lead to dramatic neurodevelopmental changes, including agenesis of the corpus callosum [36, 69, 70], developmental split-brain syndrome [69], and congenital mirror movements [37, 70, 71]. Similar noticeable changes have been described in *DCC* homozygous or haploinsufficient mice [72], highlighting the core role of *DCC* in neurodevelopmental wiring. As *DCC* expression shifts from high to low in adolescence, its functional role also shifts from broad organization of developing neuronal networks to the refinement of neuronal architecture, synaptogenesis and synaptic plasticity of established matured circuits [30, 31, 73]. Recent human studies have also shown that many polymorphisms in *DCC*, as well as altered levels of gene expression, are related to numerous neuropsychiatric conditions of developmental onset, some of which are characterized by deficits in PFC function and impulse control [30, 31]. Individual genes do not operate in isolation and cannot explain the entire spectrum of mental disorders, as it has been well established by a wealth of data from recent GWAS studies showing massive polygenicity among neuropsychiatric disorders. Therefore, *DCC* receptors act as a master organizer of specific synaptic circuits, as a part of a gene network, and we have shown that a PFC gene network for *DCC* is associated with overall brain structure [34]. Our functional analyses of the corticolimbic *DCC* gene networks suggest their implication in the development of the neural substrates underlying inhibitory control behaviors. The genes that comprise the networks are co-expressed in crucial brain regions (see Fig. 3B,  4), suggesting their spatial convergence. Furthermore, the expression of genes known to increase risk for neuropsychiatric disorders converge temporally, especially before and during the onset of the disorder [68]. Here we observed that gene expression for both networks is enriched during specific pre- and post-natal periods, including an enriched expression of the NAcc network in cortical neurons during late childhood and adolescence. As noted previously, the neurodevelopmental role that *DCC* plays changes as a function of developmental stage, and the fact that a *DCC* co-expression network is enriched again during late childhood and adolescence suggest that alterations in its function/expression can impact the adolescent development of synaptic connectivity and function in the PFC later in life.

We propose a novel type of marker for impulsivity-related phenotypes in children. Our biologically-informed approach to polygenic risk analysis aims to capture variation in the function/expression of gene networks predominantly associated with PFC and NAcc maturation, two regions subserving inhibitory control. Whether integrating relevant SNPs associated with other forms of gene expression regulation beyond cis (e.g., transcription factors, promoter regions, and chromatin modifications) in non-coding regions changes the performance of the scores, will be investigated in future studies. Exploring the association between the ePRS and inhibitory control behaviors later in life is needed in order to investigate the possible use of this genetic marker as a probabilistic risk score for vulnerability phenotypes linked to psychopathologies of adolescent onset. Our results are an example of the utility of understanding the molecular processes that govern the development of a neural circuit, and how this knowledge can be applied to predict genetic susceptibility to endophenotypes linked to psychiatric conditions.

## Supplementary information

Supplemental Information

## Data Availability

Data from MAVAN can be made available via reasonable request to the corresponding authors. For GUSTO, visit https://www.gusto.sg/. For ALSPAC, data can be purchased; the study website contains details of all the data that is available through a fully searchable data dictionary and variable search tool at http://www.bristol.ac.uk/alspac/researchers/our-data/.

## Code availability

All code used for data preparation and analysis are available upon request.
