# Supplementary material for: Corticolimbic DCC gene co-expression networks as predictors of impulsivity in children
Source: Mol Psychiatry. 2022 Apr 7;27(6):2742–50. doi: 10.1038/s41380-022-01533-7 (PMC9156406; doi:10.1038/s41380-022-01533-7)
Supplement: Supplementary file 1 — Supplemental Information [file 41380_2022_1533_MOESM1_ESM.docx]

**Corticolimbic *DCC* gene co-expression networks as predictors of impulsivity in children**

***Supplemental Information***

**Supplemental Materials and Methods**

**Participants**

We used genomic and phenotypic data from three prospective birth cohorts: 1) Maternal Adversity, Vulnerability and Neurodevelopment (MAVAN), 2) Growing Up in Singapore Towards Healthy Outcomes (GUSTO), and 3) Avon Longitudinal Study on Parents and Children (ALSPAC).

The MAVAN cohort consisted of children recruited from Montreal (Quebec) and Hamilton (Ontario), in Canada. Eligibility criteria for mothers specified being 18 years of age or older, with singleton pregnancies, and fluency in English or French. Approval for the MAVAN project was obtained from McGill University, Université de Montréal, Royal Victoria Hospital, Jewish General Hospital, Centre hospitalier de l’Université de Montréal, Hôpital Maisonneuve-Rosemount, St Joseph’s Hospital, and McMaster University. Informed consent was obtained from each participant (for more information see (1)). Extensive phenotyping was carried out from birth, including measures of reflection impulsivity assessed with the Information Sampling Task (IST) at 6 years of age. After verification of complete genotypic and phenotypic data, we retained 202 children for the current study.

The GUSTO cohort involved children born at the National University Hospital (NUH) or KK Women’s and Children’s Hospital (KKH) in Singapore, between November 2009 and May 2011. The eligibility criteria for mothers specified being of Chinese, Malay or Indian ethnicity with homogeneous parental ethnic background, as well as being aged 18 years and above at the time of recruitment. The study was approved by the National Healthcare Group Domain Specific Review Board and the Sing Health Centralized Institutional Review Board. Informed written consent was obtained from each participant (for detailed information see (2), or <https://sicsdatavault.sg/gusto/>). Extensive phenotyping was carried out from birth, including measures of impulsivity in the offspring assessed with the Stop Signal Reaction Time Task (SSRT) at 6 years of age. After verification of complete genotypic and phenotypic data, we retained 398 children in the current study.

The ALSPAC cohort consisted of children with an expected delivery date between April 1 1991, and December 31 1992, from a geographically defined area in the Southwest of England. For data collected after the age of seven, the total sample size was 15,454 pregnancies, resulting in 15,589 fetuses, of whom, 14,901 were alive at 1 year of age (for complete description see (3,4)). Phenotypic data from subjects were collected in order to assess neurodevelopment, including measures of impulsivity assessed with the SSRT task at 10 years of age. After verification of complete genotypic and phenotypic data, we retained 4392 children in the current study (detailed block diagram in Figure S1). Please note that the study website contains details of all the data that is available through a fully searchable data dictionary and variable search tool (<http://www.bristol.ac.uk/alspac/researchers/our-data/>). Ethical approval for the study was obtained from the ALSPAC Ethics and Law Committee and the Local Research Ethics Committees. A full list of the ethics committees that approved different aspects of the ALSPAC studies is available at <http://www.bristol.ac.uk/alspac/researchers/research-ethics/>. Informed consent for the use of data collected via questionnaires and clinics was obtained from participants following the recommendations of the ALSPAC Ethics and Law Committee at the time.

**Genotyping**

*MAVAN***:** Autosomal SNPs were genotyped using genome-wide platforms (PsychArray/PsychChip, Illumina) according to manufacturer’s guidelines, with genomic DNA derived from buccal epithelial cells. Quality control procedure was carried out using PLINK 1.951 (Purcell et al., 2007). Samples with a call rate less than 90% were removed. SNPs with a low call rate (< 95%), minor allele frequency (MAF) < 5%, and low p-values on Hardy-Weinberg Equilibrium exact test (p < 1e-40) were removed, which resulted in a total of 242,211 SNPs. Imputation using the Sanger Imputation Service (McCarthy et al., 2016) and the Haplotype Reference Consortium (HRC) as the reference panel (release 1.1) was performed, resulting in 20,790,893 autosomal SNPs with an info score > 0.80 (5).

*GUSTO***:** Genotyping was performed using Illumina OmniExpressExome array and split by ethnicity for quality checks. Non-autosomal SNPs, SNPs with low call rates (< 95%), MAF < 5%, and failed Hardy-Weinberg equilibrium p-value < 1e-6 were removed. Variants discordant with their respective subpopulation in the 1000 Genomes Project (6) reference panel were removed (Chinese: EAS with a threshold of 0.20; Malays: EAS with a threshold of 0.30; Indian: SAS with a threshold of 0.20). Samples with call rate < 99%, cryptic relatedness and sex/ ethnic discrepancies were excluded. The resulting data were pre-phased using SHAPEIT v2.837 with family trio information. We then used Sanger Imputation Service for imputation, choosing 1000 Genomes Project Phase 3 as reference panel and imputed “with PBWT, no pre-phasing” (the Positional Burrows Wheeler Transform algorithm) as the pipeline. Imputed data that were non-monomorphic, had biallelic SNPs and an INFO score > 0.80 were retained. Imputed genotyping data that were common in all three ethnicities (5,771,259 SNPs) were used for further analyses.

*ALSPAC.* Children were genotyped using the Illumina HumanHap550-quad chip genotyping platforms by *23andme* subcontracting the Wellcome Trust Sanger Institute, Cambridge, UK and the Laboratory Corporation of America, Burlington, NC, US. DNA was extracted from blood, cell line, and mouthwash samples, then the resulting raw genome-wide data were subjected to standard quality control methods. Participants with inconsistent self-reported and genotyped sex, minimal or excessive heterozygosity, high levels of individual missingness (>3%) and insufficient sample replication (IBD < 0.8) were excluded. SNPs with a minor allele frequency of < 1%, a call rate of < 95% or evidence for violations of Hardy-Weinberg equilibrium (p < 5e-7) were removed. Cryptic relatedness was measured as proportion of identity by descent (IBD > 0.1). Related subjects that passed all other quality control thresholds were retained during subsequent phasing and imputation. For all the subjects that were retained (N = 9115), a total of 500,527 SNPs passed these quality control filters, and after imputation with Impute v3 and Haplotype Reference Consortium (HRC) imputation reference panel (release 1.1), total genotyping data resulted in 38,898,739 SNPs. Consent for biological samples has been collected in accordance with the Human Tissue Act (2004). Informed consent for the use of data collected via questionnaires and clinics was obtained from participants following the recommendations of the ALSPAC Ethics and Law Committee at the time.

See Table S6 for a summary of the genotyping information for each cohort. See Table S7 for a brief description of the tools and datasets used throughout the study.

**Identification of corticolimbic *DCC* gene co-expression networks and ePRS calculation**

The dataset for the PFC network was generated from 29 male mice, between 10-12 weeks of age. The large-scale gene expression analysis was performed across recombinant inbred (RI) strains that were derived from the C57BL6/J x DBA/2J (BXD) genetic mapping panel, resulting in the profiling of PFC tissue from 27 BXD strains and the 2 progenitor strains (N=29). The Affymetrix Mouse Genome 430 type 2.0 microarray platform was used, and the results were normalized using the robust multi-array average (RMA) expression measure. Correlations for *Dcc* gene co-expression were calculated using the Pearson correlation coefficient, using the trait ID *1440487_at*. This dataset can be downloaded from the Gene Expression Omnibus repository (GSE28515) and can be queried on GeneNetwork (GN Accession GN135).

The dataset for the nucleus accumbens network was generated from 75 mice, 54 from the BXD panel and 21 mice reported in GeneNetwork as “other” strains. The Illumina Mouse WG-6 v1.1 Expression BeadChip platform was used, and the results were normalized using the rank invariant set normalization. Correlations for gene expression were calculated using the Pearson correlation coefficient, using the trait ID*ILM100460270*. This dataset (GeneNetwork accession GN285) does not contain information to further inspect the details of the experiment.

**Behavioral Outcomes**

We explored whether the corticolimbic *DCC*-ePRS score associates with two different aspects of impulsivity: *(i)* impulsive choice, reflecting a proneness to make risky choices, as measured by the Information Sampling Task (IST); *(ii)* impulsive action, reflecting a compromised faculty to inhibit motor responses, as measured by the stop-signal reaction time task (SSRT). In both cases, the ability to self-regulate behavior is required for interrupting or inhibiting competing inputs or actions in order to accomplish a specific goal-directed response (7) (See Figure S2).

**Information Sampling Task (IST)**. The IST is part of the CANTAB battery of neuropsychological tests and is designed to measure impulsive decision making. For each trial, a 5x5 matrix of gray boxes is presented on a computer screen, with two additional colored boxes centered below, indicating the two possible colors hidden underneath the gray boxes. Children are told that this is a game for points and that by correctly choosing the color appearing more frequently under the gray boxes they can win points. Once a grey box is selected, it immediately opens the box to reveal which of the two colors is underneath. Subjects are told that they can open as many boxes as they want, without time limitation, before making a decision. Once the subject decides and indicates which of the 2 colors appears more frequently, the color under the remaining gray boxes is revealed, along with a message stating whether the subject chose the correct response or not. The main outcome from this task is the mean probability of being correct (meanP-correct) when the response is made. Lower scores indicate more impulsivity because they are obtained when less information is gathered before deciding which color is the most prevalent (this score is a measure of impulsive choice). This task was performed by children in the MAVAN cohort at 72 months of age.

**Stop-Signal Task (SST).** The SST is designed to measure inhibitory control of a motor response, a construct of impulsive action. Participants in this task are required to respond as fast as possible to a “go” signal across many trials. On a subset of these trials, a “stop” signal is presented shortly after the “go” signal, and subjects must try to inhibit an already initiated response. We studied 2 variants of the SST:

SST – Standard: By presenting the “stop” signal with varying delays after the “go” signal, an estimate of the time required by each subject to successfully inhibit an ongoing response can be calculated. This outcome is called the Stop-signal Reaction Time or SSRT. Following each incorrect response, the subsequent presentation of the “stop” signal is delayed 50ms, while for each correctly inhibited response, the subsequent presentation of the “stop” signal is shortened by 50ms. This process serves to estimate the latency for the “stop” signal at which the participant responded correctly 50% of the time, which ultimately provides the basis for SSRT estimation. Additionally, we investigated the proportion of successfully inhibited responses when presented with the “stop” signal, which serves as another main outcome in the SST (see (8)). This task was performed by children in MAVAN and GUSTO cohorts at 6 years of age.

SST – Modified: In the modified version of the task, the “stop” signal is presented with a fixed delay after the “go” signal (see Figure S2). This modification prevents SSRT estimation, but the ability to actively suppress the response when presented with the “stop” signal is still required. Therefore, other components of the task serve as the main outcomes to study. Here, we investigated the mean reaction time of responses during unsuccessful stop trials and the proportion of unsuccessful stops. This task was performed by children in ALSPAC cohort at 10 years of age.

Note: While several measures can be calculated for the SST, we only studied estimated SSRT (for MAVAN and GUSTO), accuracy on stop trials (all 3 cohorts), and mean reaction time in incorrect stop trials (for ALSPAC).

**Validation of the PFC and NAcc *DCC* co-expression networks**

To characterize the functional and biological properties of the gene networks that comprise the corticolimbic *DCC*-ePRS score, we used 5 bioinformatic resources: 1) the STRING database ((9) <https://string-db.org/>) to construct the protein-protein interaction networks and analyze functional interactions between gene products; 2) the Cytoscape software to design and visualize the final PPI networks (10), <https://cytoscape.org/>; 3) Functional Mapping and Annotation (FUMA, (11), <https://fuma.ctglab.nl/>) to explore the expression of the genes from the co-expression networks across the 54 tissue types reported in GTEx v8; 4) MetaCore^TM^ (Clarivate Analytics) to perform enrichment analysis by mapping genes in the co-expression networks onto functional ontologies; and 5) the Cell-type Specific Expression Analysis (CSEA, (12,13) <http://genetics.wustl.edu/jdlab/csea-tool-2/>) to analyze selective enrichment of transcripts in particular brain regions and across different developmental periods (for a complete description on how to use this resource, see (12,13), and see CSEA explanation below in the supplemental methods). These different bioinformatic resources allowed us to explore the biological context in which the genes within the co-expression networks operate.

Finally, we used the human post-mortem brain samples BrainSpan dataset to evaluate expression levels of the genes comprising the PFC and the NAcc co-expression networks in childhood (n=6 for NAcc, n=12 for PFC, ages from 4 months to 11 years) and adulthood (n=7 for NAcc and PFC, ages from 19 to 40 years). This allows us to investigate whether the networks originally identified in mice are also observed in humans and the extent to which the pattern of co-expression in childhood is maintained across the lifespan. To simplify the visualization of the clustered genes and their consistency across development, we kept the same order for the genes for both time points, for each brain region. This analysis was performed using the R-based package *heatmaply* (14).

**Cell-type Specific Expression Analysis**

Results presented in Figure 3 (panel 3D) show the enrichment in gene expression in the human brain, according to region and age. The “cell-type specific expression analysis” (CSEA) tool was developed by the laboratory of Joseph Dougherty in NY (Howard Hughes Medical Institute, the Rockefeller University; see (12,13)) to investigate, among its many applications, the selective expression of genes to particular brain regions and across different developmental periods. This selective expression (termed specificity index in the tool) is calculated by comparing 60 different gene expression *profiles* – one profile for each brain region at each developmental period. For each profile, the identification of selectively enriched genes is determined at different levels of stringency (specificity index probability- pSI from 0.05 to 0.0001). A more stringent analysis results in a smaller list of enriched genes that are highly unique to that profile. Using a Fisher’s exact test (with Benjamini-Hochberg correction), this CSEA tool calculates the expected overlap between an input gene list and the previously calculated enriched lists for each of the 60 *profiles*, at 4 varying degrees of stringency (pSI = {0.05, 0.01, 0.001, 0.0001}).

**Comparison between the corticolimbic *DCC*-ePRS and other polygenic scores**

We generated other polygenic scores using our accelerated pipeline (<https://github.com/MeaneyLab/PRSoS>, (15)), for each subject. To test and compare the predictive power and functional cohesiveness of similarly large networks derived from different approaches, we calculated other polygenic scores. First, we calculated a traditional polygenic score comparable in size to the ePRS in terms of number of SNPs, considering the top 4515 SNPs from the latest ADHD GWAS, which corresponds to the GWAS *p*-value threshold 4.912e-5 (16). We investigated whether the PRS for ADHD would associate with impulsivity measurements in the same three cohorts, and then carried out a functional enrichment analysis to characterize the biological properties of the resulting group of genes that, based on genomic location, were mapped from the SNPs included in the score. We also calculated a polygenic score based on a random subset of 4515 SNPs, where we matched the proportion of SNPs from each brain region to the corticolimbic DCC-ePRS (2040 SNPs in the PFC and 2475 SNPs in the NAcc) and weighted the SNPs by the corresponding brain-region-specific effect from GTEx. We tested the association of this random ePRS with impulsivity measurements.

**Statistical Analysis**

We generated polygenic scores for all subjects with available genotypic data, with the final genetic scores categorized into low or high PRS/ePRS using a median split. All subsequent analyses, which included the comparison of baseline characteristics and the linear regressions used to examine the association of the genetic scores with the behavioral outcomes, were ran using subjects with complete genotypic and phenotypic data. Based on our inspection for influential observations, we excluded 5 datapoints from the MAVAN dataset. The population structure of the MAVAN, GUSTO, and ALSPAC cohorts were evaluated using principal component analysis of all genotyped SNPs that passed the quality control with low allele frequency (MAF > 5%) and with the following pruning parameters: not in high linkage disequilibrium (r^2^ > 0.2) across 50 kb regions (17,18) and a sliding window of 5 SNPs for MAVAN and GUSTO cohorts, and not in high linkage disequilibrium across 100 kb region, increment of 5 SNPs and variance inflation factor threshold of 1.01 for ALSPAC cohort. Based on the inspection of the scree plot, the first three principal components in MAVAN and GUSTO, and the first 10 principal components in ALSPAC, were the most informative of population structure and were included in all subsequent analyses. **Supplemental Results**

| **Enrichment Analysis (Metacore^TM^)** | | | | | | |
| --- | --- | --- | --- | --- | --- | --- |
|  |  | *PFC Network* | |  | *NAcc Network* | |
|  |  | *p-value* | *FDR* |  | *p-value* | *FDR* |
| ***Cellular Localizations*** | | | |  |  |  |
| Synapse |  | 0.000004 | 0.00024 |  | 1.301e-19 | 4.461e-17 |
| Cell junction |  | 3.304e-8 | 0.000005 |  | 1.623e-18 | 2.799e-16 |
| Postsynapse |  | 0.00016 | 0.00301 |  | 3.781e-17 | 3.542e-15 |
| Synaptic membrane |  | 0.00001 | 0.00051 |  | 4.131e-17 | 3.542e-15 |
| Asymmetric synapse |  | 0.0039 | 0.034 |  | 1.231e-15 | 8.455e-14 |
| Neuron to neuron synapse |  | 0.0059 | 0.041 |  | 3.906e-15 | 2.233e-13 |
| Postsynaptic density |  | 0.0036 | 0.032 |  | 1.448e-14 | 7.095e-13 |
| Postsynaptic specialization |  | 0.00012 | 0.0024 |  | 5.222e-14 | 2.239e-12 |
| Plasma membrane region |  | 0.00014 | 0.0024 |  | 1.307e-13 | 4.983e-12 |
| Cell periphery |  | 0.000087 | 0.00204 |  | 8.018e-13 | 2.750e-11 |
| ***Molecular Functions*** |  |  |  |  |  |  |
| Protein binding |  | 2.723e-8 | 0.0000056 |  | 2.210e-7 | 0.000092 |
| Binding |  | 0.000217 | 0.0148 |  | 0.000016 | 0.0015 |
| Voltage-gated ion channel activity |  | 0.482 | 0.634 |  | 0.000017 | 0.0015 |
| Voltage-gated channel activity |  | 0.487 | 0.643 |  | 0.000018 | 0.0015 |
| Voltage-gated cation channel activity |  | 0.675 | 0.781 |  | 0.000021 | 0.0015 |
| Protein domain specific binding |  | 0.224 | 0.403 |  | 0.000027 | 0.0016 |
| Cation channel activity |  | 0.76 | 0.83 |  | 0.000076 | 0.0038 |
| Gated channel activity |  | 0.15 | 0.32 |  | 0.000083 | 0.0038 |
| Ion channel activity |  | 0.30 | 0.48 |  | 0.000096 | 0.0040 |
| Glycosaminoglycan binding |  | 0.023 | 0.15 |  | 0.00012 | 0.0043 |
| ***Biological Processes*** |  |  |  |  |  |  |
| Modulation of chemical synaptic transmission |  | 0.0025 | 0.016 |  | 2.199e-18 | 3.724e-15 |
| Regulation of trans-synaptic signaling |  | 0.0026 | 0.016 |  | 2.349e-18 | 3.724e-15 |
| Neuron differentiation |  | 0.00021 | 0.0028 |  | 1.171e-17 | 1.138e-14 |
| Neuron development |  | 0.00093 | 0.0078 |  | 1.574e-17 | 1.138e-14 |
| Generation of neurons |  | 0.0000058 | 0.00025 |  | 1.795e-17 | 1.138e-14 |
| Trans-synaptic signaling |  | 0.000029 | 0.00075 |  | 3.090e-17 | 1.633e-14 |
| Chemical synaptic transmission |  | 0.000064 | 0.0012 |  | 1.464e-16 | 5.803e-14 |
| Anterograde trans-synaptic signaling |  | 0.000064 | 0.0012 |  | 1.464e-16 | 5.802e-14 |
| Synaptic signaling |  | 0.000091 | 0.0016 |  | 2.627e-16 | 9.256e-14 |
| Neurogenesis |  | 0.000015 | 0.00048 |  | 3.390e-16 | 1.075e-13 |

**Table S1**. Gene ontology categories related to genes included in the *DCC* gene co-expression networks in the PFC and the NAcc.


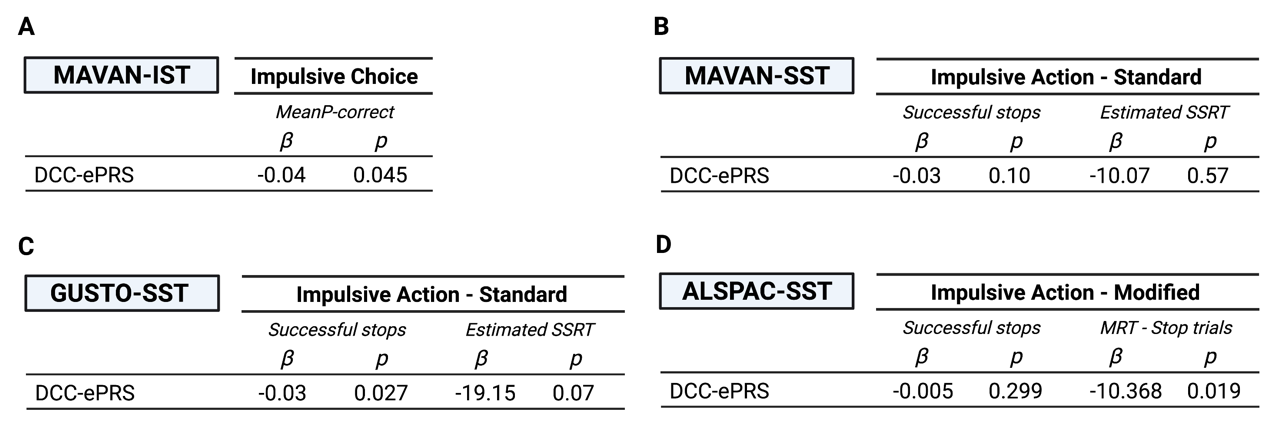


**Table S2**. Estimated effects of the ePRS on different impulsivity measures across cohorts. All models were adjusted for population stratification and sex.


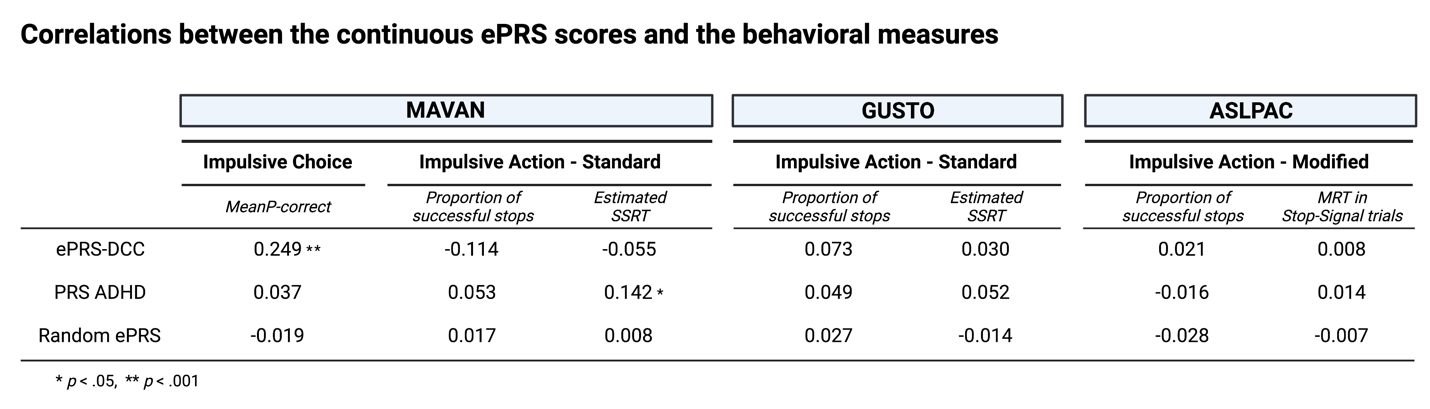


**Table S3**. Correlations between the continuous ePRS scores and the behavioral measures investigated in each cohort.

**Supplemental Figures**


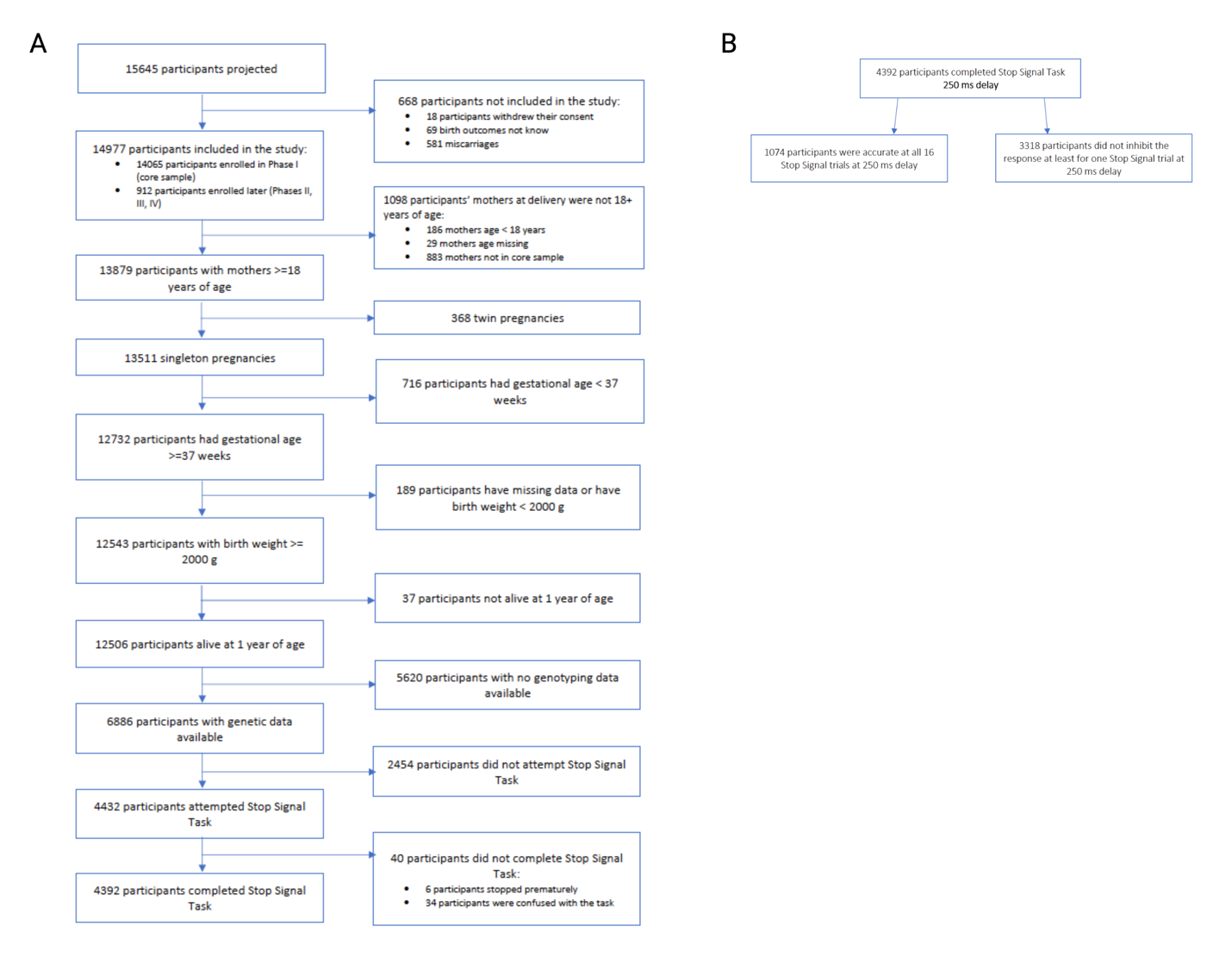


**Figure S1.** Block scheme depicting the steps involved in sample selection for ALSPAC cohort. Panel A shows a detailed scheme for the exclusion/inclusion criteria, together with the total number of participants, starting at the projected initial cohort (N=15,645) to the selection of participants that completed the SSRT task (n=4,392). Panel B shows the total number of participants for which we have proportion of successful stops and mean reaction time.


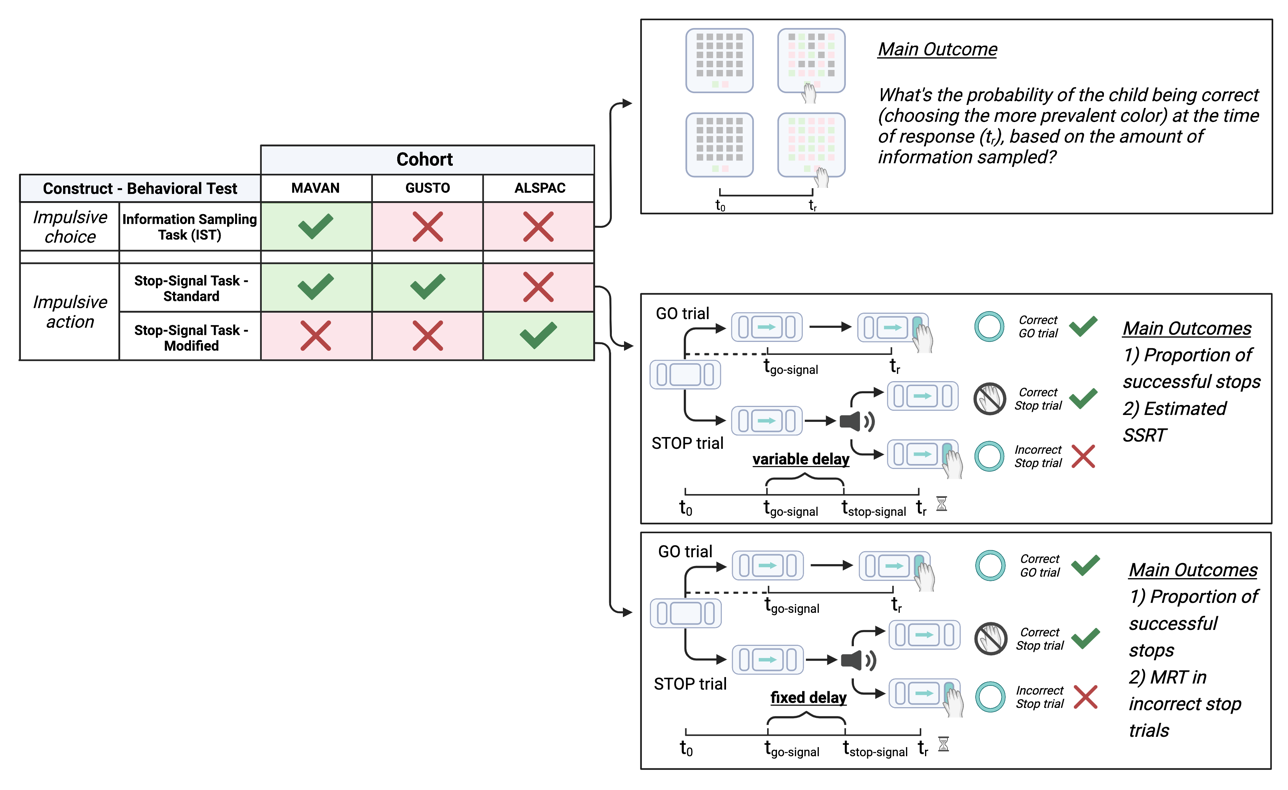


**Figure S2.** Depiction of the behavioral tasks conducted for each cohort and the main outcomes studied for each task.


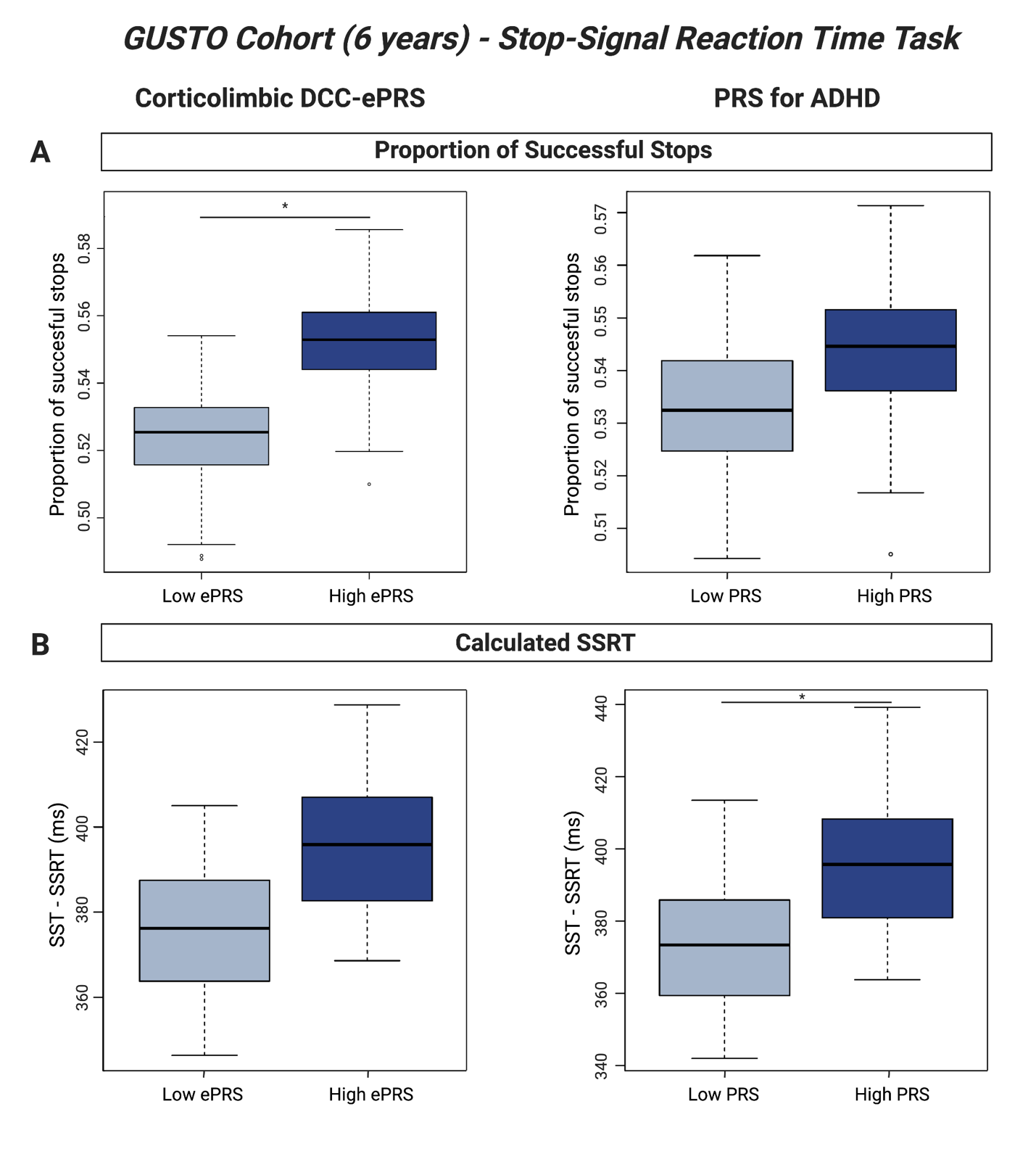


**Figure S3**. Associations between the computed genetic score for the GUSTO cohort and different components of the SSRT task, represented as boxplots. (A) Low-ePRS score group has a significantly lower proportion of successful stops when presented with the “go” signal, compared to the high-ePRS group ($\beta$ = -0.03, *p* = .027). We didn’t find a significant association between the PRS for ADHD and the proportion of successful stops ($\beta$ = -0.009, *p* = .522). (B) There is no significant association between the corticolimbic DCC-ePRS ($\beta$ = -19.152, *p* = .07) and SSRT measure, but we found that subjects with a low-PRS for ADHD had lower SSRT estimate compared to high-PRS subjects ($\beta$ = -23.865, *p* = .026). ^*^p<.05***.***

**
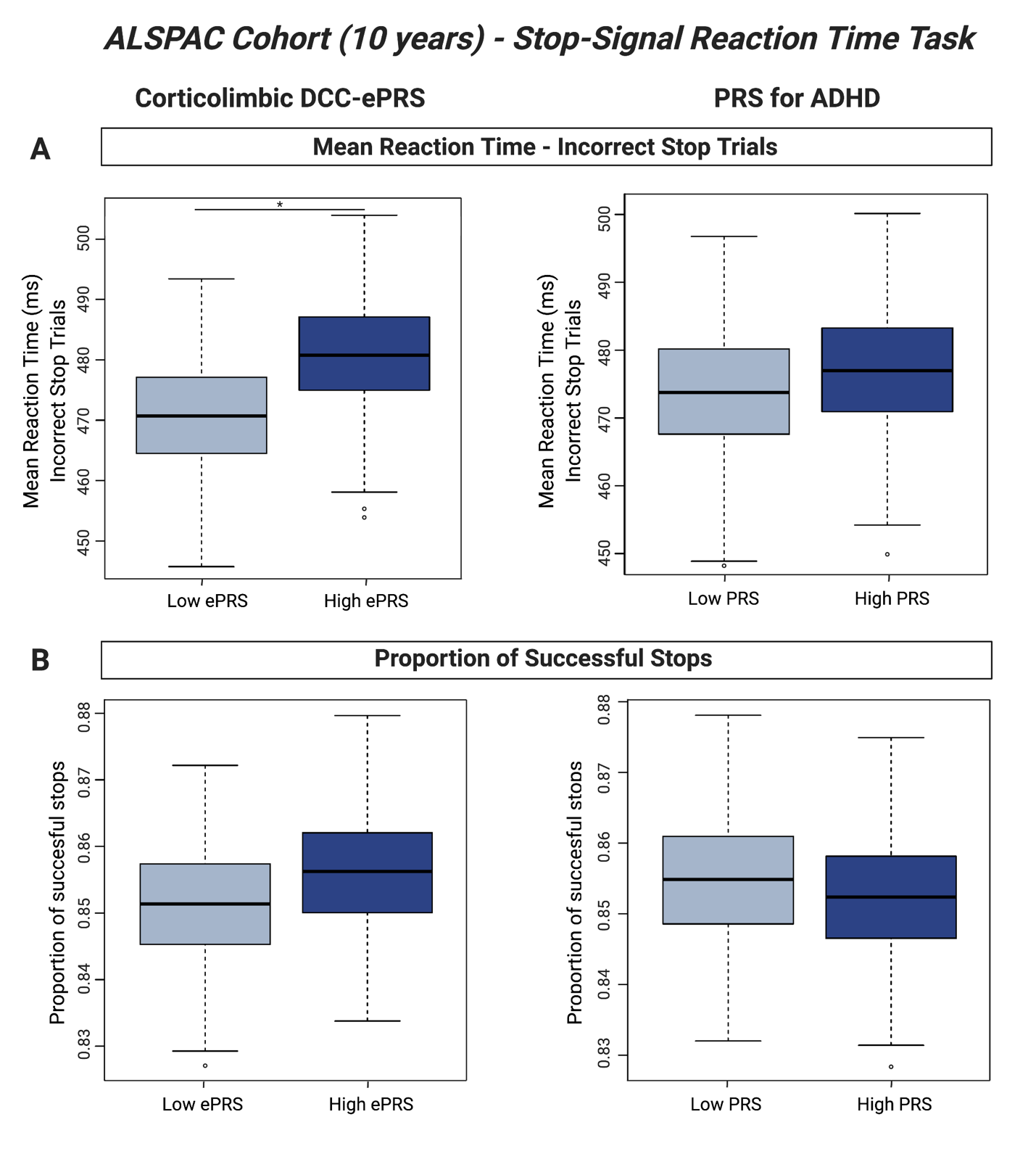
**

**Figure S4**. Associations between the computed genetic scores for the ALSPAC cohort and different measures of the SSRT task. (A) Low-ePRS score group has significantly shorter mean latency of response when presented with the “stop” signal, compared to the high-ePRS group ($\beta$ = -10.368, *p* = .019), thus showing higher levels of impulsive action. We didn’t find a significant association between the PRS for ADHD and the same outcome ($\beta$ = -3.248, *p* = .463). (B) There is no significant association between the genetic scores and the proportion of successful stops (ePRS: $\beta$ = -0.005, *p* = .299; PRS-ADHD: $\beta$ = 0.002, *p* = .657). ^*^p<.05

**
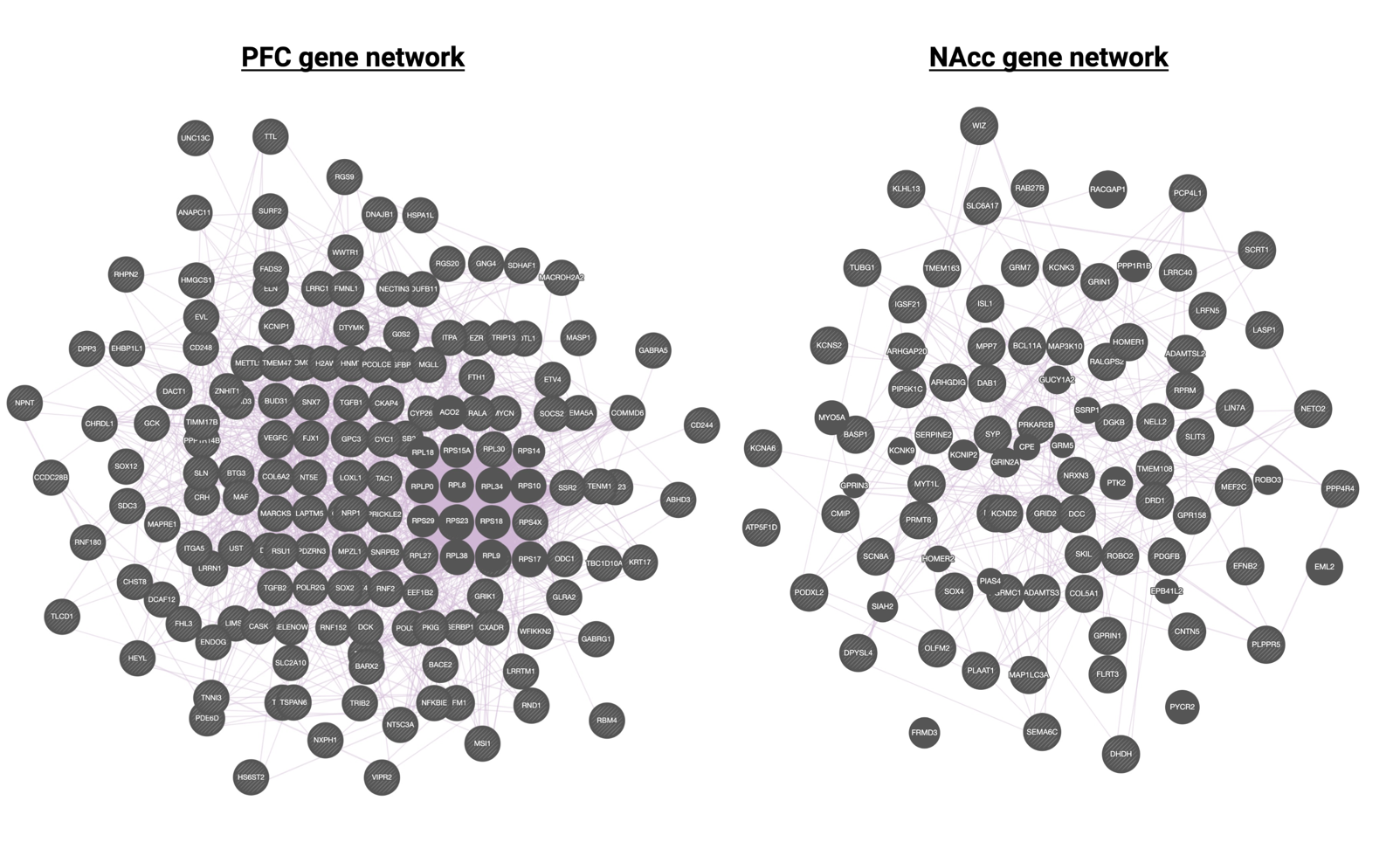
**

**Figure S5.** Gene networks for the PFC and the NAcc, created with GeneMANIA. While each gene is represented by a node, the figures clearly demonstrate the cohesiveness of both gene networks. The lines represent co-expression between genes, based on available databases on GeneMANIA.


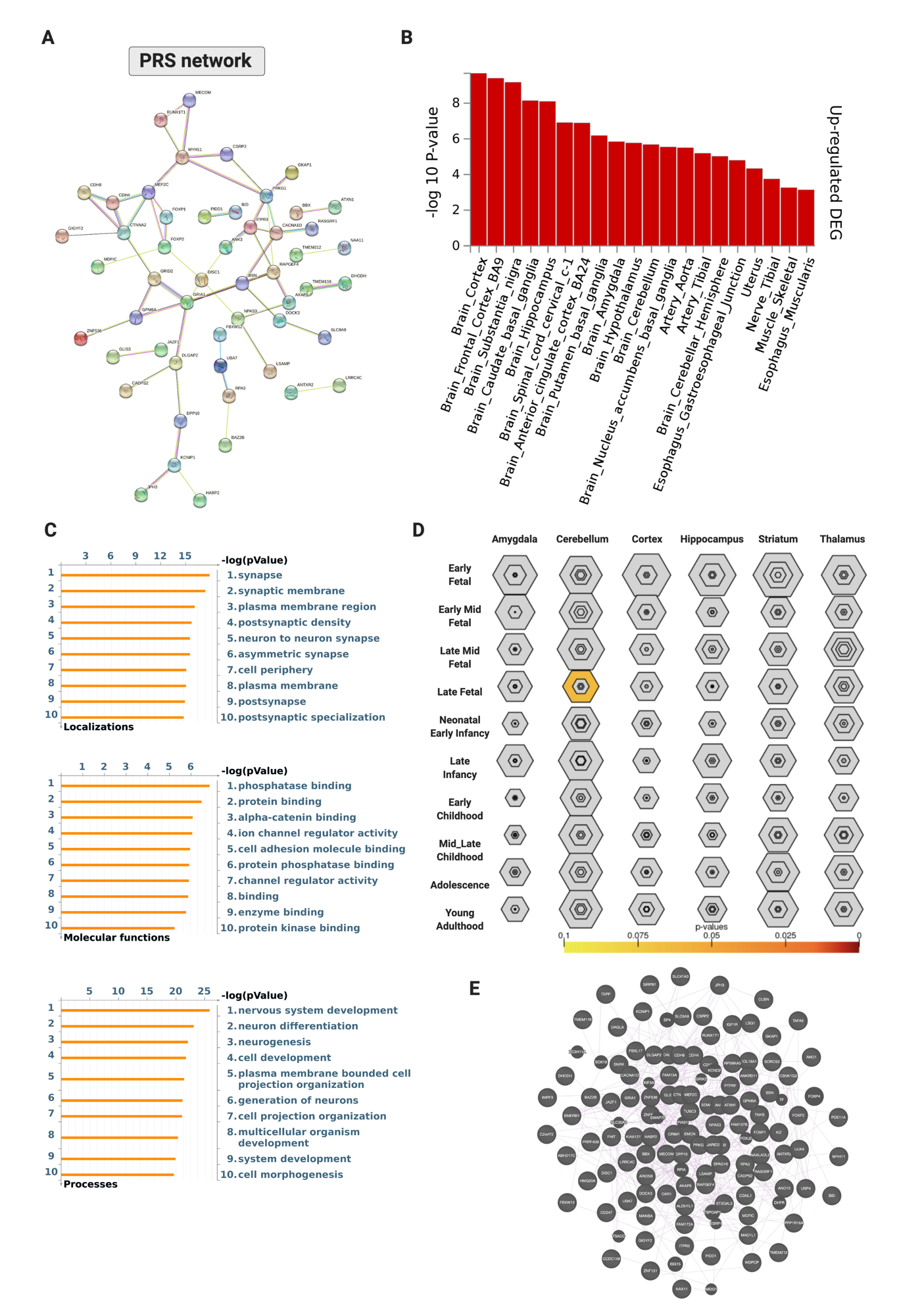


**Figure S6.** Enrichment analysis of the list of genes that make up the conventional PRS score comparable in size to the ePRS in terms of number of SNPs (n=4515, corresponding to the GWAS *p*-value threshold of 4.912e-5). (A) Protein-Protein interaction networks constructed from the PRS network. The protein network represents known functional interactions between the protein products of the genes (significant PPI enrichment). Tissue-specific gene expression analysis (B) shows that the genes that comprise the PRS are upregulated across the brain, albeit not as selectively and to a much lesser extent than the genes from the corticolimbic *DCC*-ePRS. (C) An enrichment analysis for the cellular localization, molecular functions, and biological processes of the genes in the PRS. Interestingly, these genes are enriched for similar categories across the 3 ontologies, compared to the genes in the corticolimbic *DCC* networks, indicating an enrichment for neurodevelopmental processes. (D) CSEA shows no selective spatiotemporal enrichment for the genes that comprise the PRS. (E) A depiction of the PRS gene network, created with GeneMANIA, where it’s possible to observe a highly cohesive co-expression network.

**Co-expression network in PFC**

| **Gene name** | **Ensembl ID** | **Description** |
| --- | --- | --- |
| **BTG3** | ENSG00000281484 | BTG anti-proliferation factor 3 |
| **HSPA1L** | ENSG00000236251 | heat shock protein family A (Hsp70) member 1 like |
| **CXADR** | ENSG00000154639 | CXADR Ig-like cell adhesion molecule |
| **SURF2** | ENSG00000281024 | surfeit 2 |
| **GRIK1** | ENSG00000171189 | glutamate ionotropic receptor kainate type subunit 1 |
| **SPRY2** | ENSG00000136158 | sprouty RTK signaling antagonist 2 |
| **UFM1** | ENSG00000120686 | ubiquitin fold modifier 1 |
| **GNG4** | ENSG00000282972 | G protein subunit gamma 4 |
| **SNRPB2** | ENSG00000125870 | small nuclear ribonucleoprotein polypeptide B2 |
| **METTL9** | ENSG00000284548 | methyltransferase like 9 |
| **RNF152** | ENSG00000176641 | ring finger protein 152 |
| **COL6A2** | ENSG00000142173 | collagen type VI alpha 2 chain |
| **EEF1B2** | ENSG00000283391 | eukaryotic translation elongation factor 1 beta 2 |
| **TMEM47** | ENSG00000147027 | transmembrane protein 47 |
| **GLRA2** | ENSG00000101958 | glycine receptor alpha 2 |
| **SLC2A10** | ENSG00000197496 | solute carrier family 2 member 10 |
| **TIMM17B** | ENSG00000126768 | translocase of inner mitochondrial membrane 17B |
| **BACE2** | ENSG00000182240 | beta-secretase 2 |
| **NXPH1** | ENSG00000122584 | neurexophilin 1 |
| **NRP1** | ENSG00000099250 | neuropilin 1 |
| **RSU1** | ENSG00000148484 | Ras suppressor protein 1 |
| **EFNB1** | ENSG00000090776 | ephrin B1 |
| **RPS4X** | ENSG00000198034 | ribosomal protein S4 X-linked |
| **FJX1** | ENSG00000179431 | four-jointed box kinase 1 |
| **COMMD6** | ENSG00000188243 | COMM domain containing 6 |
| **ABHD3** | ENSG00000158201 | abhydrolase domain containing 3, phospholipase |
| **GABRA5** | ENSG00000186297 | gamma-aminobutyric acid type A receptor subunit alpha5 |
| **PKIG** | ENSG00000168734 | cAMP-dependent protein kinase inhibitor gamma |
| **FTH1** | ENSG00000167996 | ferritin heavy chain 1 |
| **IGSF1** | ENSG00000147255 | immunoglobulin superfamily member 1 |
| **HS6ST2** | ENSG00000171004 | heparan sulfate 6-O-sulfotransferase 2 |
| **LRRN1** | ENSG00000175928 | leucine rich repeat neuronal 1 |
| **GPC3** | ENSG00000147257 | glypican 3 |
| **TRIP13** | ENSG00000071539 | thyroid hormone receptor interactor 13 |
| **SEMA5A** | ENSG00000112902 | semaphorin 5A |
| **MAPRE1** | ENSG00000101367 | microtubule associated protein RP/EB family member 1 |
| **ITPA** | ENSG00000125877 | inosine triphosphatase |
| **TAC1** | ENSG00000006128 | tachykinin precursor 1 |
| **CYP26A1** | ENSG00000095596 | cytochrome P450 family 26 subfamily A member 1 |
| **LAPTM5** | ENSG00000162511 | lysosomal protein transmembrane 5 |
| **RPL23** | ENSG00000125691 | ribosomal protein L23 |
| **SDC3** | ENSG00000162512 | syndecan 3 |
| **CHST8** | ENSG00000124302 | carbohydrate sulfotransferase 8 |
| **KRT17** | ENSG00000128422 | keratin 17 |
| **MYCN** | ENSG00000134323 | MYCN proto-oncogene, bHLH transcription factor |
| **EVL** | ENSG00000196405 | Enah/Vasp-like |
| **ROMO1** | ENSG00000125995 | reactive oxygen species modulator 1 |
| **ETV4** | ENSG00000175832 | ETS variant transcription factor 4 |
| **ITGA5** | ENSG00000161638 | integrin subunit alpha 5 |
| **PDZRN3** | ENSG00000121440 | PDZ domain containing ring finger 3 |
| **TBC1D10A** | ENSG00000099992 | TBC1 domain family member 10A |
| **BUD31** | ENSG00000106245 | BUD31 homolog |
| **EHBP1L1** | ENSG00000173442 | EH domain binding protein 1 like 1 |
| **NFKBIE** | ENSG00000146232 | NFKB inhibitor epsilon |
| **MAF** | ENSG00000178573 | MAF bZIP transcription factor |
| **RHPN2** | ENSG00000131941 | rhophilin Rho GTPase binding protein 2 |
| **SLN** | ENSG00000170290 | sarcolipin |
| **DAB2** | ENSG00000153071 | DAB adaptor protein 2 |
| **FHL3** | ENSG00000183386 | four and a half LIM domains 3 |
| **CHST11** | ENSG00000171310 | carbohydrate sulfotransferase 11 |
| **DCK** | ENSG00000156136 | deoxycytidine kinase |
| **RPL30** | ENSG00000156482 | ribosomal protein L30 |
| **RPL12** | ENSG00000197958 | ribosomal protein L12 |
| **LOXL1** | ENSG00000129038 | lysyl oxidase like 1 |
| **NDUFB11** | ENSG00000147123 | NADH:ubiquinone oxidoreductase subunit B11 |
| **WFIKKN2** | ENSG00000173714 | WAP, follistatin/kazal, immunoglobulin, kunitz and netrin domain containing 2 |
| **SOX12** | ENSG00000177732 | SRY-box transcription factor 12 |
| **TSPAN6** | ENSG00000000003 | tetraspanin 6 |
| **BARX2** | ENSG00000043039 | BARX homeobox 2 |
| **COTL1** | ENSG00000103187 | coactosin like F-actin binding protein 1 |
| **MARCKS** | ENSG00000277443 | myristoylated alanine rich protein kinase C substrate |
| **PRICKLE2** | ENSG00000163637 | prickle planar cell polarity protein 2 |
| **HMGCS1** | ENSG00000112972 | 3-hydroxy-3-methylglutaryl-CoA synthase 1 |
| **UST** | ENSG00000111962 | uronyl 2-sulfotransferase |
| **RGS20** | ENSG00000147509 | regulator of G protein signaling 20 |
| **RNF180** | ENSG00000164197 | ring finger protein 180 |
| **RGS9** | ENSG00000108370 | regulator of G protein signaling 9 |
| **SOCS2** | ENSG00000120833 | suppressor of cytokine signaling 2 |
| **CKAP4** | ENSG00000136026 | cytoskeleton associated protein 4 |
| **RPS3** | ENSG00000149273 | ribosomal protein S3 |
| **GABRG1** | ENSG00000163285 | gamma-aminobutyric acid type A receptor subunit gamma1 |
| **PPP1R14B** | ENSG00000173457 | protein phosphatase 1 regulatory inhibitor subunit 14B |
| **CHRDL1** | ENSG00000101938 | chordin like 1 |
| **CHCHD3** | ENSG00000106554 | coiled-coil-helix-coiled-coil-helix domain containing 3 |
| **ZNHIT1** | ENSG00000106400 | zinc finger HIT-type containing 1 |
| **TNNI3** | ENSG00000129991 | troponin I3, cardiac type |
| **CASK** | ENSG00000147044 | calcium/calmodulin dependent serine protein kinase |
| **LRRC17** | ENSG00000128606 | leucine rich repeat containing 17 |
| **VIPR2** | ENSG00000106018 | vasoactive intestinal peptide receptor 2 |
| **SOX2** | ENSG00000181449 | SRY-box transcription factor 2 |
| **PRDX4** | ENSG00000123131 | peroxiredoxin 4 |
| **CRH** | ENSG00000147571 | corticotropin releasing hormone |
| **SNX7** | ENSG00000162627 | sorting nexin 7 |
| **DACT1** | ENSG00000165617 | dishevelled binding antagonist of beta catenin 1 |
| **MSI1** | ENSG00000135097 | musashi RNA binding protein 1 |
| **VEGFC** | ENSG00000150630 | vascular endothelial growth factor C |
| **DCAF12** | ENSG00000198876 | DDB1 and CUL4 associated factor 12 |
| **RALA** | ENSG00000006451 | RAS like proto-oncogene A |
| **HNMT** | ENSG00000150540 | histamine N-methyltransferase |
| **TTL** | ENSG00000114999 | tubulin tyrosine ligase |
| **IGFBP5** | ENSG00000115461 | insulin like growth factor binding protein 5 |
| **CD248** | ENSG00000174807 | CD248 molecule |
| **CYC1** | ENSG00000179091 | cytochrome c1 |
| **EZR** | ENSG00000092820 | ezrin |
| **DLX1** | ENSG00000144355 | distal-less homeobox 1 |
| **SDHAF1** | ENSG00000205138 | succinate dehydrogenase complex assembly factor 1 |
| **ENDOG** | ENSG00000167136 | endonuclease G |
| **ELN** | ENSG00000049540 | elastin |
| **POU3F3** | ENSG00000198914 | POU class 3 homeobox 3 |
| **MPZL1** | ENSG00000197965 | myelin protein zero like 1 |
| **PCOLCE** | ENSG00000106333 | procollagen C-endopeptidase enhancer |
| **DNAJB1** | ENSG00000132002 | DnaJ heat shock protein family (Hsp40) member B1 |
| **RNF2** | ENSG00000121481 | ring finger protein 2 |
| **POLR2G** | ENSG00000168002 | RNA polymerase II subunit G |
| **NPNT** | ENSG00000168743 | nephronectin |
| **RPL27** | ENSG00000131469 | ribosomal protein L27 |
| **DTYMK** | ENSG00000168393 | deoxythymidylate kinase |
| **KCNIP1** | ENSG00000182132 | potassium voltage-gated channel interacting protein 1 |
| **WWTR1** | ENSG00000018408 | WW domain containing transcription regulator 1 |
| **TGFB2** | ENSG00000092969 | transforming growth factor beta 2 |
| **TGFB1** | ENSG00000105329 | transforming growth factor beta 1 |
| **DPP3** | ENSG00000254986 | dipeptidyl peptidase 3 |
| **WSB2** | ENSG00000176871 | WD repeat and SOCS box containing 2 |
| **G0S2** | ENSG00000123689 | G0/G1 switch 2 |
| **UNC13C** | ENSG00000137766 | unc-13 homolog C |
| **GCK** | ENSG00000106633 | glucokinase |
| **CD244** | ENSG00000122223 | CD244 molecule |
| **PDE6D** | ENSG00000156973 | phosphodiesterase 6D |
| **TLCD1** | ENSG00000160606 | TLC domain containing 1 |
| **MASP1** | ENSG00000127241 | mannan binding lectin serine peptidase 1 |
| **SSR2** | ENSG00000163479 | signal sequence receptor subunit 2 |
| **NT5E** | ENSG00000135318 | 5'-nucleotidase ecto |
| **RND1** | ENSG00000172602 | Rho family GTPase 1 |
| **FADS2** | ENSG00000134824 | fatty acid desaturase 2 |
| **FMNL1** | ENSG00000184922 | formin like 1 |
| **RBM4** | ENSG00000173933 | RNA binding motif protein 4 |
| **ANAPC11** | ENSG00000141552 | anaphase promoting complex subunit 11 |
| **MGLL** | ENSG00000074416 | monoglyceride lipase |
| **ODC1** | ENSG00000115758 | ornithine decarboxylase 1 |
| **HEYL** | ENSG00000163909 | hes related family bHLH transcription factor with YRPW motif like |
| **LRRTM1** | ENSG00000162951 | leucine rich repeat transmembrane neuronal 1 |
| **SERBP1** | ENSG00000142864 | SERPINE1 mRNA binding protein 1 |
| **CCDC28B** | ENSG00000160050 | coiled-coil domain containing 28B |
| **TRIB2** | ENSG00000071575 | tribbles pseudokinase 2 |
| **LIMS2** | ENSG00000072163 | LIM zinc finger domain containing 2 |
| **H2AW** | ENSG00000284841 | H2A.W histone |
| **TENM1** | ENSG00000009694 | teneurin transmembrane protein 1 |
| **TAFA1** | ENSG00000183662 | TAFA chemokine like family member 1 |
| **NECTIN3** | ENSG00000177707 | nectin cell adhesion molecule 3 |
| **SELENOW** | ENSG00000178980 | selenoprotein W |
| **MACROH2A2** | ENSG00000099284 | macroH2A.2 histone |
| **NT5C3A** | ENSG00000122643 | 5'-nucleotidase, cytosolic IIIA |

**Table S4.** Genes co-expressed with *DCC* in the PFC

**Co-expression network in NAcc**

| **Gene name** | **Ensembl ID** | **Description** |
| --- | --- | --- |
| **NEU4** | ENSG00000277926 | neuraminidase 4 |
| **PPP4R4** | ENSG00000278326 | protein phosphatase 4 regulatory subunit 4 |
| **MAGI1** | ENSG00000282956 | membrane associated guanylate kinase, WW and PDZ domain containing 1 |
| **PRKAR2B** | ENSG00000284096 | protein kinase cAMP-dependent type II regulatory subunit beta |
| **FLRT3** | ENSG00000125848 | fibronectin leucine rich transmembrane protein 3 |
| **DCC** | ENSG00000187323 | DCC netrin 1 receptor |
| **RAB27B** | ENSG00000041353 | RAB27B, member RAS oncogene family |
| **SCRT1** | ENSG00000284923 | scratch family transcriptional repressor 1 |
| **PDGFB** | ENSG00000100311 | platelet derived growth factor subunit B |
| **EFNB2** | ENSG00000125266 | ephrin B2 |
| **MAP1LC3A** | ENSG00000101460 | microtubule associated protein 1 light chain 3 alpha |
| **KCNA6** | ENSG00000151079 | potassium voltage-gated channel subfamily A member 6 |
| **ARHGDIG** | ENSG00000242173 | Rho GDP dissociation inhibitor gamma |
| **PGRMC1** | ENSG00000101856 | progesterone receptor membrane component 1 |
| **SOX4** | ENSG00000124766 | SRY-box transcription factor 4 |
| **KLHL13** | ENSG00000003096 | kelch like family member 13 |
| **GRM7** | ENSG00000196277 | glutamate metabotropic receptor 7 |
| **NETO2** | ENSG00000171208 | neuropilin and tolloid like 2 |
| **ISL1** | ENSG00000016082 | ISL LIM homeobox 1 |
| **ROBO2** | ENSG00000185008 | roundabout guidance receptor 2 |
| **KCNK3** | ENSG00000171303 | potassium two pore domain channel subfamily K member 3 |
| **ADAMTSL2** | ENSG00000197859 | ADAMTS like 2 |
| **LASP1** | ENSG00000002834 | LIM and SH3 protein 1 |
| **CNTN5** | ENSG00000149972 | contactin 5 |
| **COL5A1** | ENSG00000130635 | collagen type V alpha 1 chain |
| **DPYSL4** | ENSG00000151640 | dihydropyrimidinase like 4 |
| **MAP3K10** | ENSG00000130758 | mitogen-activated protein kinase kinase kinase 10 |
| **GPR158** | ENSG00000151025 | G protein-coupled receptor 158 |
| **KCND2** | ENSG00000184408 | potassium voltage-gated channel subfamily D member 2 |
| **ADAMTS3** | ENSG00000156140 | ADAM metallopeptidase with thrombospondin type 1 motif 3 |
| **KCNS2** | ENSG00000156486 | potassium voltage-gated channel modifier subfamily S member 2 |
| **SYP** | ENSG00000102003 | synaptophysin |
| **GRID2** | ENSG00000152208 | glutamate ionotropic receptor delta type subunit 2 |
| **BCL11A** | ENSG00000119866 | BAF chromatin remodeling complex subunit BCL11A |
| **HOMER1** | ENSG00000152413 | homer scaffold protein 1 |
| **PODXL2** | ENSG00000114631 | podocalyxin like 2 |
| **BASP1** | ENSG00000176788 | brain abundant membrane attached signal protein 1 |
| **GRIN1** | ENSG00000176884 | glutamate ionotropic receptor NMDA type subunit 1 |
| **IGSF21** | ENSG00000117154 | immunoglobin superfamily member 21 |
| **SKIL** | ENSG00000136603 | SKI like proto-oncogene |
| **SLC6A17** | ENSG00000197106 | solute carrier family 6 member 17 |
| **NRXN3** | ENSG00000021645 | neurexin 3 |
| **DRD1** | ENSG00000184845 | dopamine receptor D1 |
| **DHDH** | ENSG00000104808 | dihydrodiol dehydrogenase |
| **TMEM163** | ENSG00000152128 | transmembrane protein 163 |
| **LRFN5** | ENSG00000165379 | leucine rich repeat and fibronectin type III domain containing 5 |
| **MEF2C** | ENSG00000081189 | myocyte enhancer factor 2C |
| **SLIT3** | ENSG00000184347 | slit guidance ligand 3 |
| **DGKB** | ENSG00000136267 | diacylglycerol kinase beta |
| **NELL2** | ENSG00000184613 | neural EGFL like 2 |
| **MPP7** | ENSG00000150054 | membrane palmitoylated protein 7 |
| **RPRM** | ENSG00000177519 | reprimo, TP53 dependent G2 arrest mediator homolog |
| **CMIP** | ENSG00000153815 | c-Maf inducing protein |
| **GPRIN1** | ENSG00000169258 | G protein regulated inducer of neurite outgrowth 1 |
| **WIZ** | ENSG00000011451 | WIZ zinc finger |
| **PIP5K1C** | ENSG00000186111 | phosphatidylinositol-4-phosphate 5-kinase type 1 gamma |
| **LRRC40** | ENSG00000066557 | leucine rich repeat containing 40 |
| **TMEM108** | ENSG00000144868 | transmembrane protein 108 |
| **MYT1L** | ENSG00000186487 | myelin transcription factor 1 like |
| **SCN8A** | ENSG00000196876 | sodium voltage-gated channel alpha subunit 8 |
| **OLFM2** | ENSG00000105088 | olfactomedin 2 |
| **LIN7A** | ENSG00000111052 | lin-7 homolog A, crumbs cell polarity complex component |
| **PCP4L1** | ENSG00000248485 | Purkinje cell protein 4 like 1 |
| **SERPINE2** | ENSG00000135919 | serpin family E member 2 |
| **TUBG1** | ENSG00000131462 | tubulin gamma 1 |
| **SEMA6C** | ENSG00000143434 | semaphorin 6C |
| **ARHGAP20** | ENSG00000137727 | Rho GTPase activating protein 20 |
| **PRMT6** | ENSG00000198890 | protein arginine methyltransferase 6 |
| **DAB1** | ENSG00000173406 | DAB adaptor protein 1 |
| **RALGPS2** | ENSG00000116191 | Ral GEF with PH domain and SH3 binding motif 2 |
| **PLPPR5** | ENSG00000117598 | phospholipid phosphatase related 5 |
| **TENM3** | ENSG00000218336 | teneurin transmembrane protein 3 |
| **PLAAT1** | ENSG00000127252 | phospholipase A and acyltransferase 1 |
| **ATP5F1D** | ENSG00000099624 | ATP synthase F1 subunit delta |

**Table S5.** Genes co-expressed with *DCC* in the NAcc


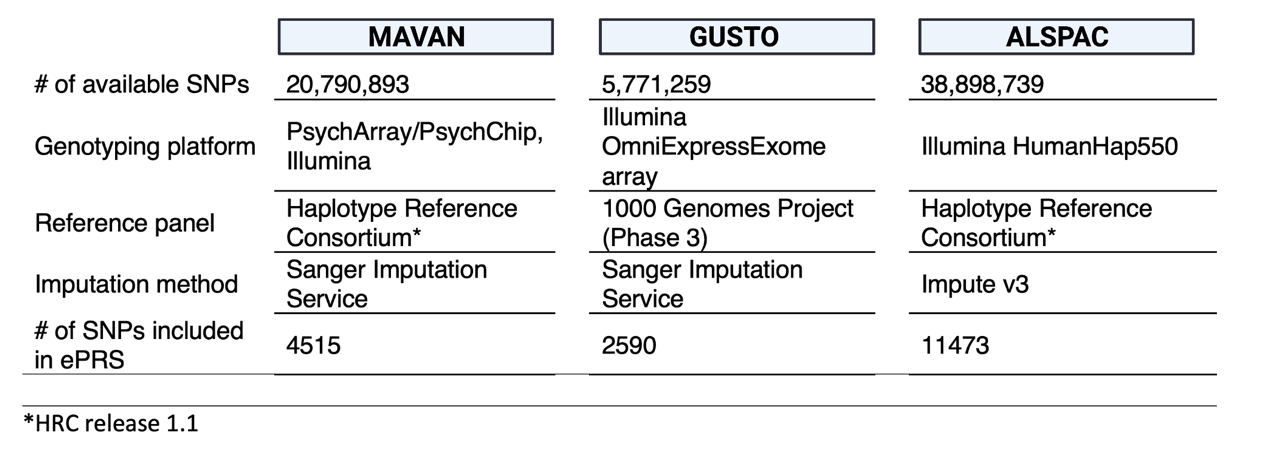


**Table S6.** Genotyping information for the 3 cohorts


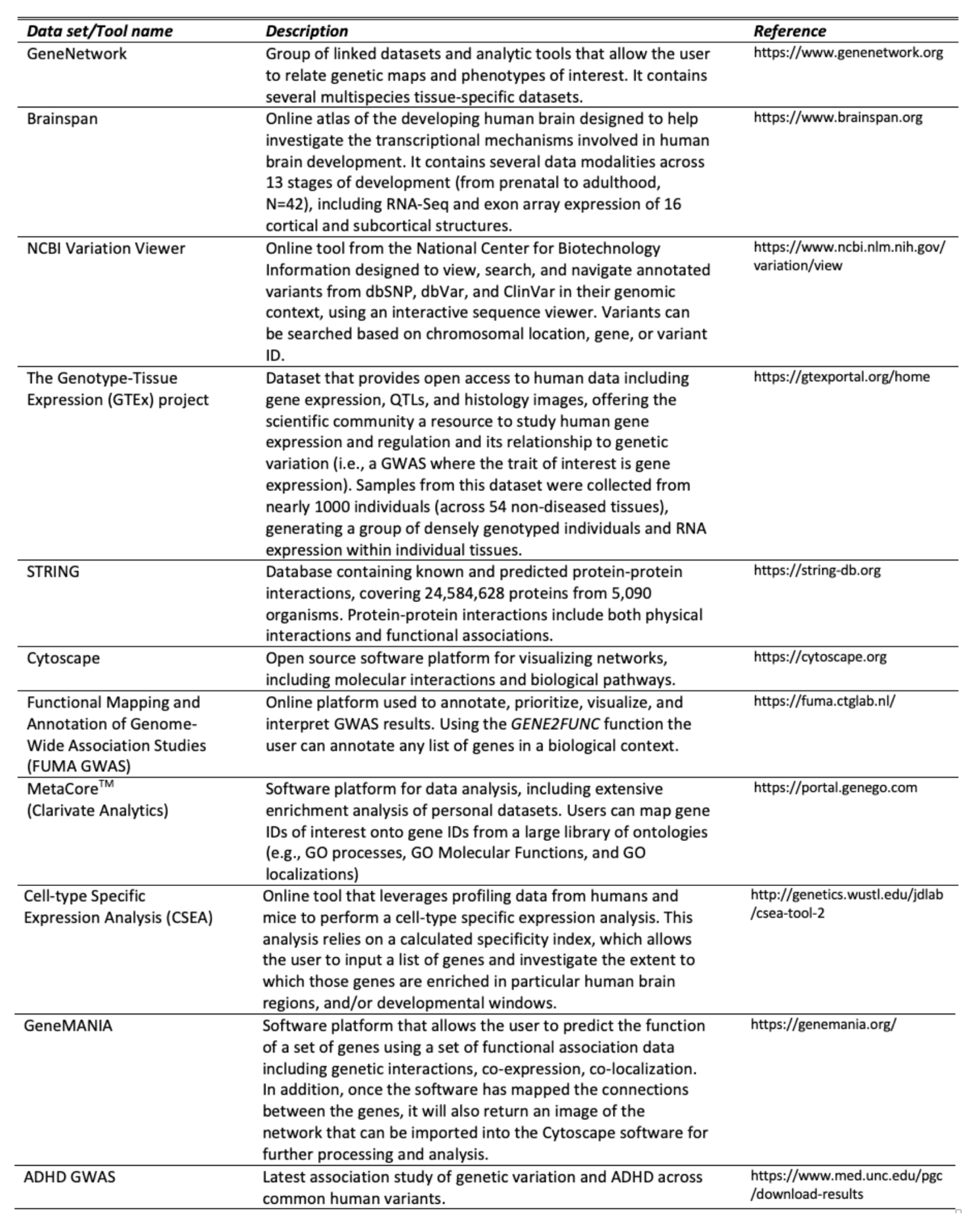


**Table S7.** Description of the datasets and tools used throughout the study

**Supplemental References**

1. O’Donnell KA, Gaudreau H, Colalillo S, Steiner M, Atkinson L, Moss E, et al. The maternal adversity, vulnerability and neurodevelopment project: Theory and methodology. Can J Psychiatry. 2014;59(9):497–508.

2. Soh S-E, Tint MT, Gluckman PD, Godfrey KM, Rifkin-Graboi A, Chan YH, et al. Cohort Profile: Growing Up in Singapore Towards healthy Outcomes (GUSTO) birth cohort study. Int J Epidemiol. 2014 Oct 1;43(5):1401–9.

3. Boyd A, Golding J, Macleod J, Lawlor DA, Fraser A, Henderson J, et al. Cohort profile: The ’Children of the 90s’-The index offspring of the avon longitudinal study of parents and children. Int J Epidemiol. 2013 Feb 1;42(1):111–27.

4. Fraser A, Macdonald-wallis C, Tilling K, Boyd A, Golding J, Davey smith G, et al. Cohort profile: The avon longitudinal study of parents and children: ALSPAC mothers cohort. Int J Epidemiol. 2013 Feb 1;42(1):97–110.

5. McCarthy S, Das S, Kretzschmar W, Delaneau O, Wood AR, Teumer A, et al. A reference panel of 64,976 haplotypes for genotype imputation. Nat Genet. 2016;48(10):1279–83.

6. Auton A, Abecasis GR, Altshuler DM, Durbin RM, Abecasis GR, Bentley DR, et al. A global reference for human genetic variation. Nature. 2015 Oct 1;526(7571):68–74.

7. Somerville LH, Casey BJ. Developmental neurobiology of cognitive control and motivational systems. Curr Opin Neurobiol. 2010;20(2):236–41.

8. Verbruggen F, Aron AR, Band GPH, Beste C, Bissett PG, Brockett AT, et al. A consensus guide to capturing the ability to inhibit actions and impulsive behaviors in the stop-signal task. Elife. 2019;8:1–26.

9. Szklarczyk D, Gable AL, Lyon D, Junge A, Wyder S, Huerta-Cepas J, et al. STRING v11: Protein-protein association networks with increased coverage, supporting functional discovery in genome-wide experimental datasets. Nucleic Acids Res. 2019;47(D1):D607–13.

10. Demchak B, Hull T, Reich M, Liefeld T, Smoot M, Ideker T, et al. Cytoscape: the network visualization tool for GenomeSpace workflows. F1000Research. 2014;2014:1–12.

11. Watanabe K, Taskesen E, Van Bochoven A, Posthuma D. Functional mapping and annotation of genetic associations with FUMA. Nat Commun. 2017 Dec 28;8(1):1826.

12. Dougherty JD, Schmidt EF, Nakajima M, Heintz N. Analytical approaches to RNA profiling data for the identification of genes enriched in specific cells. Nucleic Acids Res. 2010;38(13):4218–30.

13. Xu X, Wells AB, O’Brien DR, Nehorai A, Dougherty JD. Cell type-specific expression analysis to identify putative cellular mechanisms for neurogenetic disorders. J Neurosci. 2014;34(4):1420–31.

14. Galili T, O’callaghan A, Sidi J, Sievert C. Heatmaply: an R package for creating interactive cluster heatmaps for online publishing. Bioinformatics. 2018;34(9):1600–2.

15. Chen LM, Yao N, Garg E, Zhu Y, Nguyen TTT, Pokhvisneva I, et al. PRS-on-Spark (PRSoS): A novel, efficient and flexible approach for generating polygenic risk scores. BMC Bioinformatics. 2018 Dec 8;19(1):295.

16. Demontis D, Walters RK, Martin J, Mattheisen M, Als TD, Agerbo E, et al. Discovery of the first genome-wide significant risk loci for attention deficit/hyperactivity disorder. Nat Genet. 2019;51(1):63–75.

17. Price AL, Patterson NJ, Plenge RM, Weinblatt ME, Shadick NA, Reich D. Principal components analysis corrects for stratification in genome-wide association studies. Nat Genet. 2006;38(8):904–9.

18. Wray NR, Lee SH, Mehta D, Vinkhuyzen AAE, Dudbridge F, Middeldorp CM. Research Review: Polygenic methods and their application to psychiatric traits. Vol. 55, Journal of Child Psychology and Psychiatry and Allied Disciplines. Blackwell Publishing Ltd; 2014. p. 1068–87.
